# Paradoxical Activation of Entheseal Myeloid Cells by JAK1 and Tyk2 Inhibitors via Interleukin‐10 Antagonism

**DOI:** 10.1002/art.43210

**Published:** 2025-07-04

**Authors:** Sami Giryes, Chi Wong, Charlie Bridgewood, Mark Harland, Ala Altaie, Nicole McDermott, Kerem Abacar, Abhay Rao, Almas Khan, Tristan McMillan, Peter Loughenbury, Robert Dunsmuir, Vishal Borse, Tom Macleod, Dennis McGonagle

**Affiliations:** ^1^ Leeds Institute of Rheumatic and Musculoskeletal Medicine, University of Leeds, Leeds, United Kingdom, and B. Shine Rheumatology Institute, Rambam Healthcare Campus Haifa Israel; ^2^ Leeds Institute of Rheumatic and Musculoskeletal Medicine, University of Leeds Leeds United Kingdom; ^3^ Leeds Biomedical Research Centre, Leeds Teaching Hospitals, National Institute for Health and Care Research Leeds United Kingdom; ^4^ Leeds Institute of Rheumatic and Musculoskeletal Medicine, University of Leeds and Leeds Biomedical Research Centre, Leeds Teaching Hospitals, National Institute for Health and Care Research Leeds United Kingdom

## Abstract

**Objective:**

JAK inhibition (JAKi) is effective in seronegative spondyloarthropathy (SpA) spectrum disorders, but Tyk2 inhibition failed in SpA spectrum ulcerative colitis, and tofacitinib showed minimal benefit in Crohn disease, which highlights the complex role for JAK/STAT signaling in different inflammatory processes. In this study, we investigated whether JAKi might paradoxically activate entheseal innate immunity and aimed to identify the key regulatory cytokines involved in this process.

**Methods:**

Spinal entheseal tissue was activated with Toll‐like receptor (TLR) agonists, including TLR4 and interleukin‐1 (IL‐1) family proteins, and entheseal T cells were activated with anti‐CD3/anti‐CD28 with IL‐23/IL‐1β. JAKi via upadacitinib (JAK1/JAK2), deucravacitinib (Tyk2), and ritlecitinib (JAK3) inhibition was evaluated using multiplex cytokine assays, intracellular flow, and bulk RNA sequencing (RNAseq) and cytokine blocking or stimulation.

**Results:**

Following interferon γ stimulation, JAK1 inhibition blocked STAT1 phosphorylation in entheseal cells and strongly blocked activated entheseal T cell tumor necrosis factor α (TNFα), IL‐17A, and IL‐17F production. The opposite effect was evident in entheseal myeloid cell with exaggerated TLR4 and other adjuvant‐mediated cytokine production including IL‐23 (~10‐fold increase; *P* < 0.001) or TNFα (~10‐fold increase; *P* < 0.0001). This myeloid effect was induced by upadacitinib and deucravacitinib but not ritlecitinib, suggesting IL‐10R JAK1/Tyk2 signaling. Bulk RNAseq showed a multifaceted impact of JAKi on myeloid activation with strong M1 type monocyte polarization under TLR4 stimulation and JAK1 inhibition confirmed by flow cytometry. Direct IL‐10 inhibition recapitulated inflammatory cytokine elevations and IL‐10R agonist largely, but not completely, rescued this phenotype.

**Conclusion:**

These findings help explain the emergent efficacy of Tyk2 blockade in SpA spectrum–related arthritis that is not IL‐10 dependent but indicates why such strategies may not be a panacea for SpA spectrum disorder–related intestinal inflammation.

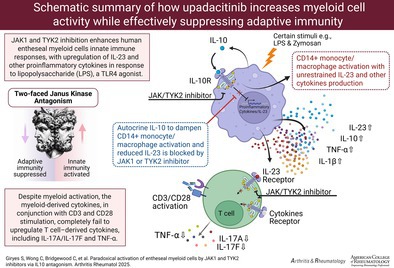

## INTRODUCTION

Spondyloarthropathy (SpA) encompasses ankylosing spondylitis (AS), psoriatic arthropathy (PsA), and related psoriasis; reactive arthritis; and inflammatory bowel disease (IBD)–associated arthritis evident in Crohn disease (CD) and ulcerative colitis (UC). These disorders are associated with enthesitis, which is the cardinal SpA lesion, and associated with both tumor necrosis factor (TNF) and interleukin‐23 (IL‐23)/IL‐17 pathway dysregulation at the synovio‐entheseal complex leading to joint inflammation.^1^, ^2^, ^3^

Cytokine signaling through the JAK‐STAT pathway activates more than 50 soluble factors including cytokines, interferons (IFNs), and endocrine factors.^3^, ^4^, ^5^ The JAK and STAT families include four JAK members (JAK1, JAK2, JAK3, and Tyk2) and seven STAT members.^5^ IL‐23 is a critical cytokine in the pathogenesis of SpA, being immunogenetically linked to all SpA spectrum manifestations^2^, ^6^, ^7^ and to type‐17 T cell production pathogenic disease driver cytokines including IL‐17A, IL‐17F, TNF, and others.^2^, ^8^

Currently, three JAK inhibitors (JAKi) are licensed or soon to be adopted in SpA‐associated arthropathy including tofacitinib (considered a pan‐JAKi), upadacitinib (predominantly JAK1 inhibition, with reports of JAK2 inhibition in certain assays ranging from 2‐ to 60‐fold less selectivity than JAK1) and deucravacitinib (highly selective Tyk2 inhibition).^9^, ^10^, ^11^, ^12^, ^13^, ^14^ The exact mechanism of JAKi efficacy in SpA is unclear because the pivotal IL‐17A, IL‐17F, and TNF cytokines do not signal directly via the JAK pathway.^15^, ^16^ The IL‐23 signaling is mediated by JAK2‐Tyk2 and subsequent STAT3 phosphorylation veering lymphoid cells toward IL‐17 production.^17^, ^18^ A proposed mechanisms JAKi efficacy in SpA is blocking IL‐23–dependent JAK2‐Tyk2 signaling.^4^, ^19^

However, JAKi is not a panacea for all SpA‐related, spectrum‐associated inflammation including IBD, which contrasts to TNF monoclonal antibody inhibition that treats all joint and intestinal manifestations. For example, tofacitinib has minimal CD efficacy and consequently is not licensed for that indication.^20^ Furthermore, in contrast to IL‐23 blockers, Tyk2 inhibition with deucravacitinib failed to show efficacy in UC despite good efficacy for both psoriasis and PsA,^21^ although JAK1 inhibition that blocks the common γ chain needed for T cell proliferation and activation is license in IBD. Of note, IL‐10 is a key cytokine linked to inborn errors of innate immunity with early onset IBD but is not linked to SpA spectrum skeletal inflammation.^22^ Furthermore, IL‐10 signals via JAK1 and^23^ Tyk2 and could thus be a pivotal cytokine in potential differences between enthesis and intestinal immunology across the SpA spectrum.

Upadacitinib, a JAK1 inhibitor (2‐ to 60‐fold selective over JAK2, >100‐fold selective over JAK3, and does not inhibit Tyk2),^12^, ^14^, ^24^ has proven efficacy in AS, PsA, UC, and, recently, CD.^25^, ^26^, ^27^, ^28^, ^29^ Given the differential efficacy for JAKi across SpA spectrum disorders and IBD, we explored the impact of JAKi on both enthesis innate and adaptive immune responses to better understand the emerging reverse translational immunology pertaining to JAKi in SpA spectrum disorders.

## MATERIALS AND METHODS

Peripheral blood and normal spinous process enthesis were obtained from 32 donors with no systemic autoimmune or inflammatory diseases who were undergoing spinal decompression or surgery for scoliosis correction of thoracic or lumbar vertebrae using methodology previously described.^30^ Written informed consent to participate was given by all patients before taking part, and research was conducted in compliance with the Helsinki Declaration. Entheseal sample collection was approved by the Northwest‐Greater Manchester West Research Ethics Committee (REC:16/NW/0797). Patients and/or the public were not involved in the design, conduct, reporting, or dissemination plans of this research. Data are available on reasonable request.

The collected enthesis samples were separated into soft tissue and peri‐entheseal bone (PEB) with subsequent PEB minced into small fragments ≤5 mm^2^, followed by mechanical digestion with cells collected after phosphate buffered saline (PBS) washing. Thereafter, cells were strained and red cells lysis was performed using the ammonium chloride method with two PBS washing steps and resuspension in RPMI 1640 (10% fetal bovine serum, 10 U/mL of penicillin and streptomycin). T cells were further enriched from entheseal cells with pan T cell isolation kit (Miltenyi). Peripheral blood leukocytes were isolated using red cell lysis with ammonium chloride. Three PBS washes were performed before resuspension in RPMI 1640.

Isolated cells were plated at 5 × 10^5^ cells per well in 96 well plates in RPMI 1640. For T cell stimulation, 96 well plates were coated with 1 μg/mL of anti‐CD3 (Life Technologies, orthoclone T 3) before cell seeding and stimulation with 2.5 μg/mL of anti‐CD28 with or without the addition of 25 ng/mL of IL‐1β and IL‐23. For upadacitinib inhibition of T cell activation, cells were pretreated with varying upadacitinib (indicated in the Results section) concentrations in a separate well for one hour before transfer to an anti‐CD3–coated 96 well plate and subsequent stimulation. For myeloid activation, cells were pretreated with or without upadacitinib (concentration indicated in the Results section) for one hour before stimulation with either lipopolysaccharide (LPS) (10 ng/mL), IL‐1β (25 ng/mL), IL‐36α (100 ng/mL), mannan (10 μg/mL), zymosan (10 μg/mL), or IL‐10 (concentration indicated in the Results section). THP‐1 cell lines were cultured in RPMI 1640 and plated at 2 × 10^5^ cells per well in 96 well plates before stimulation.

For cytokine blocking experiments, neutralizing antibodies anti–IL‐10Rα, anti–IL‐19, and anti‐IFN α/β Receptor 1 (IFNAR1) (Supplementary Table S1) were added to cells at the concentrations, indicated in the Results section, one hour before stimulation. NF‐κB inhibition experiments conducted on peripheral blood leukocytes used the specific inhibitor caffeic acid phenethyl ester at 1 μ*M* added to cells three hours after LPS stimulation.^31^ Following stimulation, cells were pelleted by centrifugation and supernatants collected for cytokine analysis. Cells were collected for further analysis by flow cytometry or isolation of RNA.

### Luciferase assay

THP‐1 cells stably transduced with an NF‐κB–driven luciferase reporter were pretreated with 1 μ*M* of upadacitinib one hour before stimulation with 10 ng/mL LPS. Luciferase activity was then measured each hour after LPS stimulation (0–5 hours). Luciferase activity was measured by the addition of Dual‐Glo substrate and buffer solution (Promega) according to the manufacturer's instructions, allowing 20 minutes of incubation time (room temperature), followed by measurement of luminosity by a Cytation 5 multimode plate reader (Agilent BioTek).

### Flow cytometry

For intracellular analysis of cytokines, cells were treated with GolgiPlug (BD Biosciences; 1 μL per 10^6^ cells) eight hours before analysis. Cells were stained for cell viability (Zombie Fixable Dyes, BioLegend, 1:1,000, 20‐minute incubation) before blocking with 10% mouse serum and 1% human IgG for 10 minutes. Cells were then stained with surface marker antibodies (Supplementary Table S1) at concentrations indicated by the manufacturers for 30 minutes in staining buffer (PBS, 1 m*M* EDTA, 2% bovine serum albumin with BD brilliant stain buffer). For analysis of intracellular cytokines, after surface staining cells were fixed and permeabilized using a BD Cytofix/Cytoperm fixation/permeabilization kit according to the manufacturer's instructions and then stained with intracellular cytokine antibodies for 30 minutes (Supplementary Table S1). Stained cells were analyzed on a Beckman‐Coulter Cytoflex LX cytometer.

For analysis of phosphorylated STAT1, cells were fixed in 4% paraformaldehyde for 10 minutes at 37°C immediately after stimulation. Cells were then permeabilized using BD Phosflow Perm Buffer III for 30 minutes on ice. After fixation, cells were resuspended in staining buffer and anti‐pSTAT1 (Supplementary Table S1) for one hour. Cells were analyzed on a Beckman‐Coulter Cytoflex LX cytometer.

### Bulk RNA sequencing and analysis

RNA was extracted from cell pellets (~5 × 10^5^ cells) using the Total RNA Purification Kit (Norgen Biotek). Sequencing libraries were prepared, and sequenced (PE150, 9 Gb per sample) on the Illumina NovaSeq X plus at Novogene (Novogene). Following quality control (removal of low‐quality reads and reads containing adapters and poly‐N), reads were aligned to the reference genome using Hisat 2 version 2.0.5. featureCounts version 1.5.0‐p3 was used to count reads and calculate fragments per Kb of transcript per million bp sequenced. Differential expression analysis was performed with DESeq2 (DESeq2RPackage 1.20.0), and adjusted *P* values calculated using the Benjamin and Hochberg's approach. Genes with a adjusted *P* value ≤0.05 were assigned as differentially expressed. The clusterProfiler R package was used to test the statistical enrichment of differentially expressed genes in Kyoto Encyclopedia of Genes and Genomes (KEGG) pathways, with pathways with corrected *P* values <0.05 considered significantly enriched. Gene set enrichment analysis (GSEA) for KEGG pathways was performed using the GSEA analysis tool (http://broadinstitute.org/gsea/index.jsp).

### Cytokine measurement and statistical analysis

Cytokines were measured either by enzyme‐linked immunosorbent assay (ELISA) (IL‐23, TNFα; Thermofisher) or by bead‐based multiplexed immunoassay (LEGENDplexTM, BioLegend) according to the manufacturer's instructions. GraphPad Prism software was employed with analysis of variance employment to calculate significance and Dunnet's test used for multiple comparisons. Specific statistical tests are described in the corresponding figure legends. Significant differences between control and test with *P* values less than 0.05 were denoted with an asterisk, as indicated in the figure legends, or labeled with the exact *P* value when they were greater than 0.05 but there were trends evident. Any specific statistical tests are outlined in the figure legends.

## RESULTS

### JAKi strongly inhibiting entheseal T cell activity

**Figure 1:**
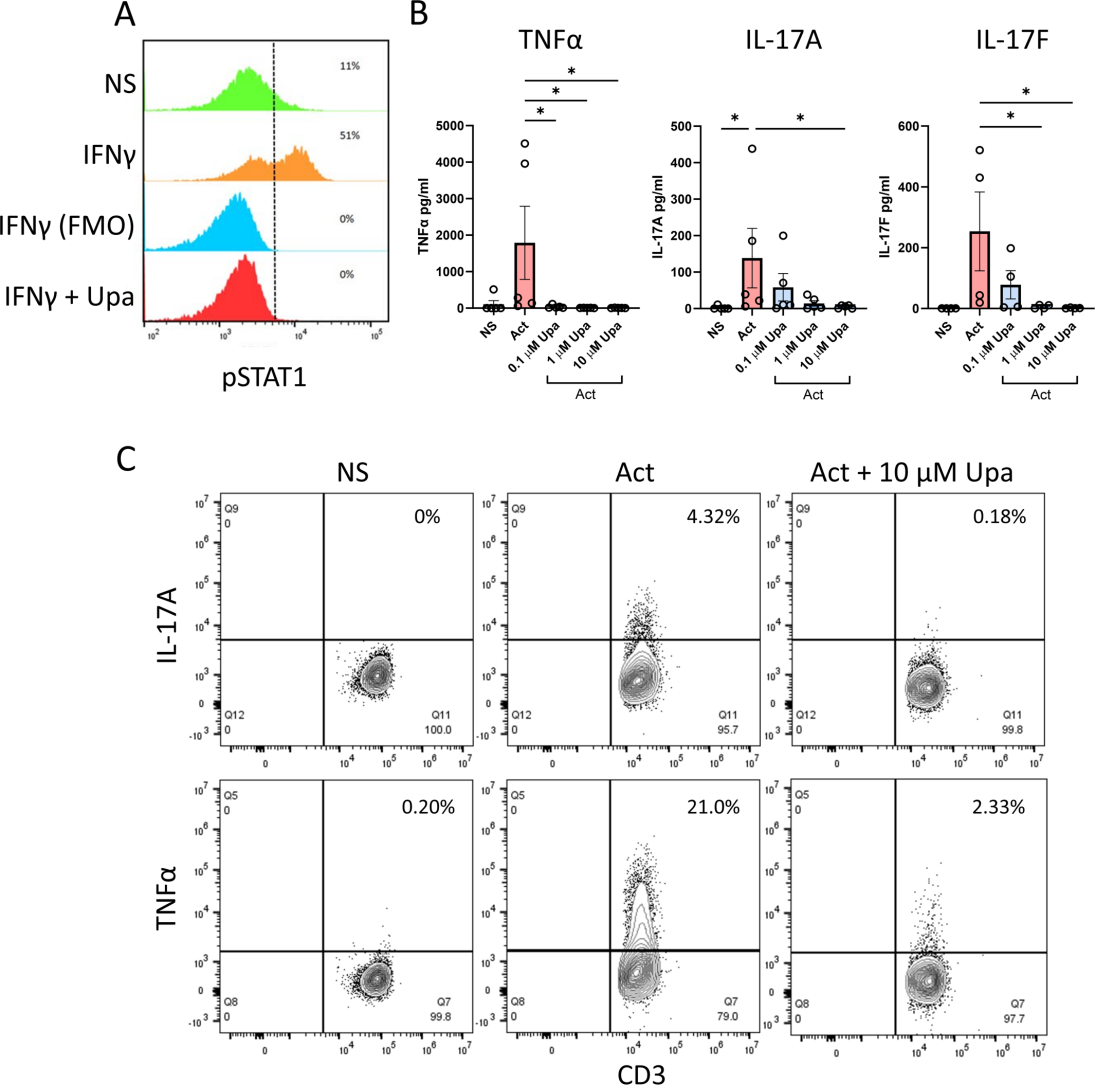
JAK1 inhibition strongly inhibits entheseal T cell activity. (A) Isolated entheseal cells untreated or stimulated with IFNγ (20 ng/mL; 15 minutes) with or without Upa (10 μ*M*) and pSTAT1 measured by flow cytometry. (B) Isolated entheseal cells stimulated with anti‐CD3 (1 μg/mL), anti‐CD28 (2.5 μg/mL), IL‐1β, and IL‐23 (25 ng/mL each) for 48 hours for T cell activation with and without indicated concentrations of upadacitinib. Secreted cytokines TNFα (n = 5), IL‐17A (n = 5), and IL‐17F (n = 4) were measured by enzyme‐linked immunosorbent assay. (C) Enriched CD3^+^ entheseal cells were treated as in panel B and analyzed for intracellular IL‐17A and TNFα by flow cytometry. Gating strategy is shown in Supplementary Figure S1. Dot plots show CD45^+^CD3^+^ gated population (gating strategy Supplementary Figure S1). Flow cytometry (A and C) is representative of three independent experiments. *P < 0.05 (statistical test), data are presented as means with SEMs (B). Act, activation; FMO, fluorescence minus one; IFNγ, interferon γ; IL‐17A, interleukin 17A; NS, untreated entheseal cells; pSTAT1, phosphorylated STAT1; TNFα, tumor necrosis factor α; Upa, upadacitinib.

We initially evaluated upadacitinib as an exemplar of JAKi with broad efficacy across SpA and IBD. Entheseal leukocytes were pretreated with upadacitinib and then stimulated with IFNγ for 15 minutes. IFNγ signaling requires activation of JAK1 and JAK2 to facilitate STAT1 phosphorylation, which allows translocation to the nucleus and subsequent gene transcription.^32^ Analysis by intracellular flow cytometry demonstrated upadacitinib effectively prevented phosphorylation of STAT1 (Figure 1A).

To evaluate upadacitinib effects on T cell–derived SpA‐driving cytokines TNFα and IL‐17, entheseal leukocytes were pretreated with upadacitinib at concentrations representative of patient sera levels over the course of a dose (0.1–10 μ*M*),^33^ then stimulated for 48 hours with type‐17‐driving cytokines (IL‐23 and IL‐1β) and anti‐CD3/CD28 antibodies for T cell receptor (TCR)–dependent activation of T cells with upadacitinib significantly down‐regulating TNFα, IL‐17A, and IL‐17F production from activated T cells, thus showing how JAKi block activation of entheseal derived T cells (Figure 1B). Intracellular flow cytometry on CD3‐enriched entheseal T cells treated in the same conditions showed reduction in TNFα^+^ and IL‐17A^+^ CD3^+^ cells after TCR activation when pretreated with upadacitinib (Figure 1C; gating strategy shown in Supplementary Figure S1).

### Upadacitinib up‐regulating CD14 ^+^ entheseal cell IL‐23 and TNFα induced via Toll‐like receptor activation

**Figure 2:**
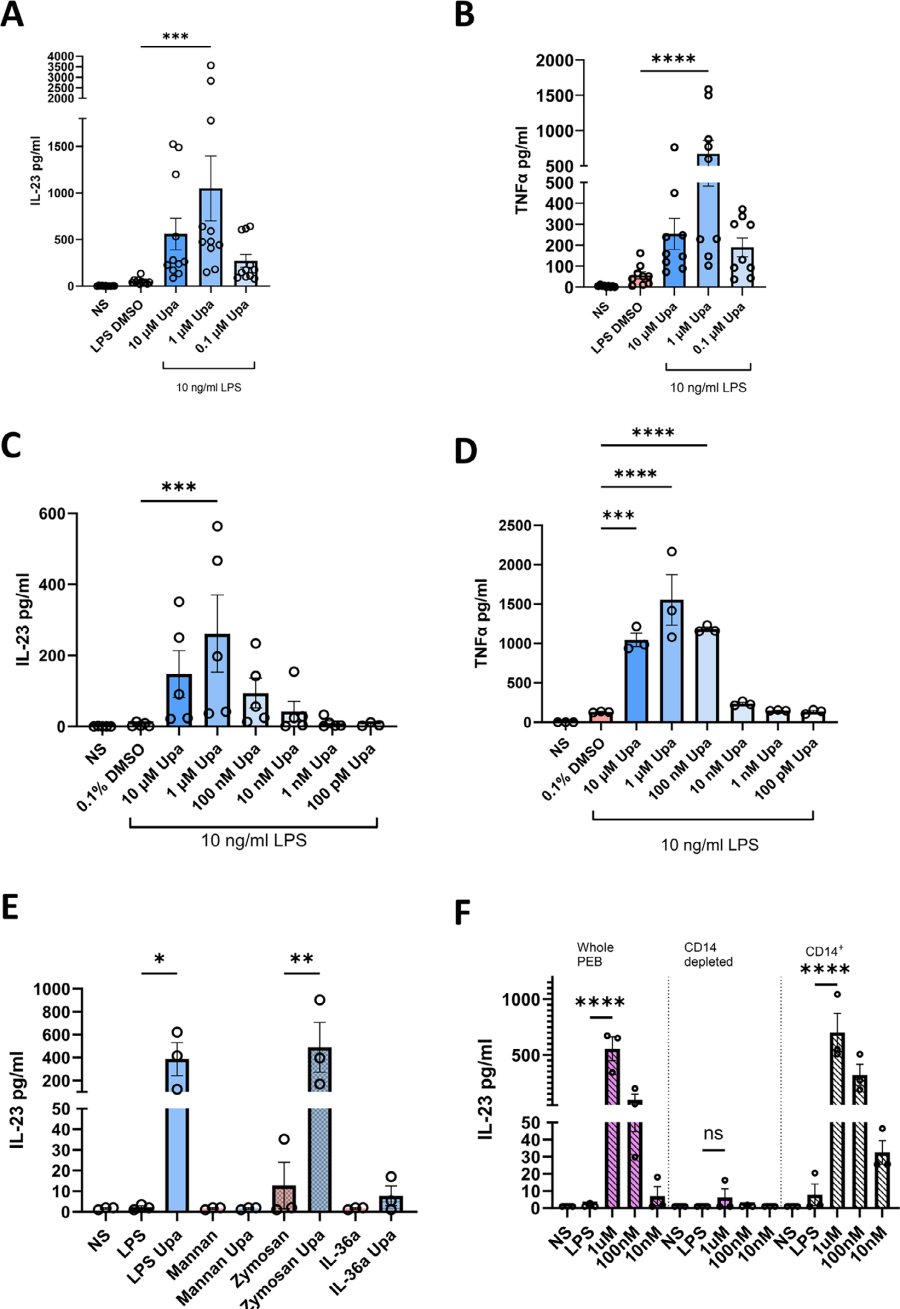
JAK1 inhibition increases NF‐κB–driven IL‐23 and TNFα from CD14^+^ entheseal cells. Entheseal cells untreated or stimulated with LPS (10 ng/mL) with an indicated concentration of Upa for 24 hours and (A and C) IL‐23 and (B and D) TNFα measured by enzyme‐linked immunosorbent assay (ELISA). (E) Entheseal cells stimulated with LPS, mannan, zymosan, IL‐1β, IL‐36α with and without 1 μ*M* Upa, and IL‐23 measured by ELISA. (F) Peripheral blood leukocytes, CD14^+^ depleted peripheral blood leukocytes, or isolated CD14^+^ cells were treated with 10 ng/mL LPS with or without Upa. Figures are representative of (A) 11, (B) 9, (C) 5, or (D–E) 3 independent experiments. *P < 0.05, **P < 0.01, ***P < 0.001, ****P < 0.0001 (statistical test). Data are presented as means with SEMs. IL‐23, interleukin 23; LPS, lipopolysaccharide; NS, untreated entheseal cells; PEB, peri‐entheseal bone; TNFα, tumor necrosis factor α; Upa, upadacitinib. Color figure can be viewed in the online issue, which is available at http://onlinelibrary.wiley.com/doi/10.1002/art.43210/abstract.

Entheseal leukocytes were pretreated with upadacitinib (0.1–10 μ*M*) before stimulation with LPS (10 ng/mL) to evaluate the impact of JAKi on IL‐23 and TNF production. Following 24‐hour stimulation, IL‐23 and TNFα supernatants were measured using ELISA. Upadacitinib addition strongly up‐regulated LPS‐induced IL‐23 and TNFα production, with approximately 10‐ to 20‐fold increased levels observed at 1 μ*M* upadacitinib pretreatment (Figure 2A and B). Because these results showed an increase in cytokine production even at 0.1 μ*M*, we extended the range of upadacitinib down to 100 p*M*. As shown in Figure 2C and D, the effect is strongest at 1 μ*M*, yet it is still evident at 10 n*M*.

To determine whether IL‐23 and TNFα up‐regulation was unique to LPS stimulation, we tested other pathogen‐associated molecular patterns and inflammatory cytokines. Zymosan and 1 μ*M* of upadacitinib also further increased IL‐23 production (*P* < 0.01), and a nonsignificant increased trend when stimulated with IL‐36γ, demonstrating this effect is beyond LPS (Figure 2E). All stimuli tested that exhibit this effect (Figure 2E) signal via NF‐κB suggesting a common mechanism for myeloid cell activation.

We have previously shown entheseal monocytes are important producers of IL‐23 following LPS stimulation and therefore performed isolation and depletion of CD14^+^ monocytes from entheseal isolates.^34^ As shown in Figure 2F, depletion of CD14^+^ monocytes ablated the upadacitinib‐induced IL‐23 level enhancement, and isolated CD14^+^ cells strongly exhibited IL‐23 up‐regulation after LPS stimulation following pretreatment with upadacitinib (Figure 2F), suggesting that upadacitinib‐induced up‐regulation of IL‐23 and TNFα production was monocyte lineage cells.

### Transcriptional analysis implicating IL‐10 signaling in JAKi‐induced myeloid activation

**Figure 3:**
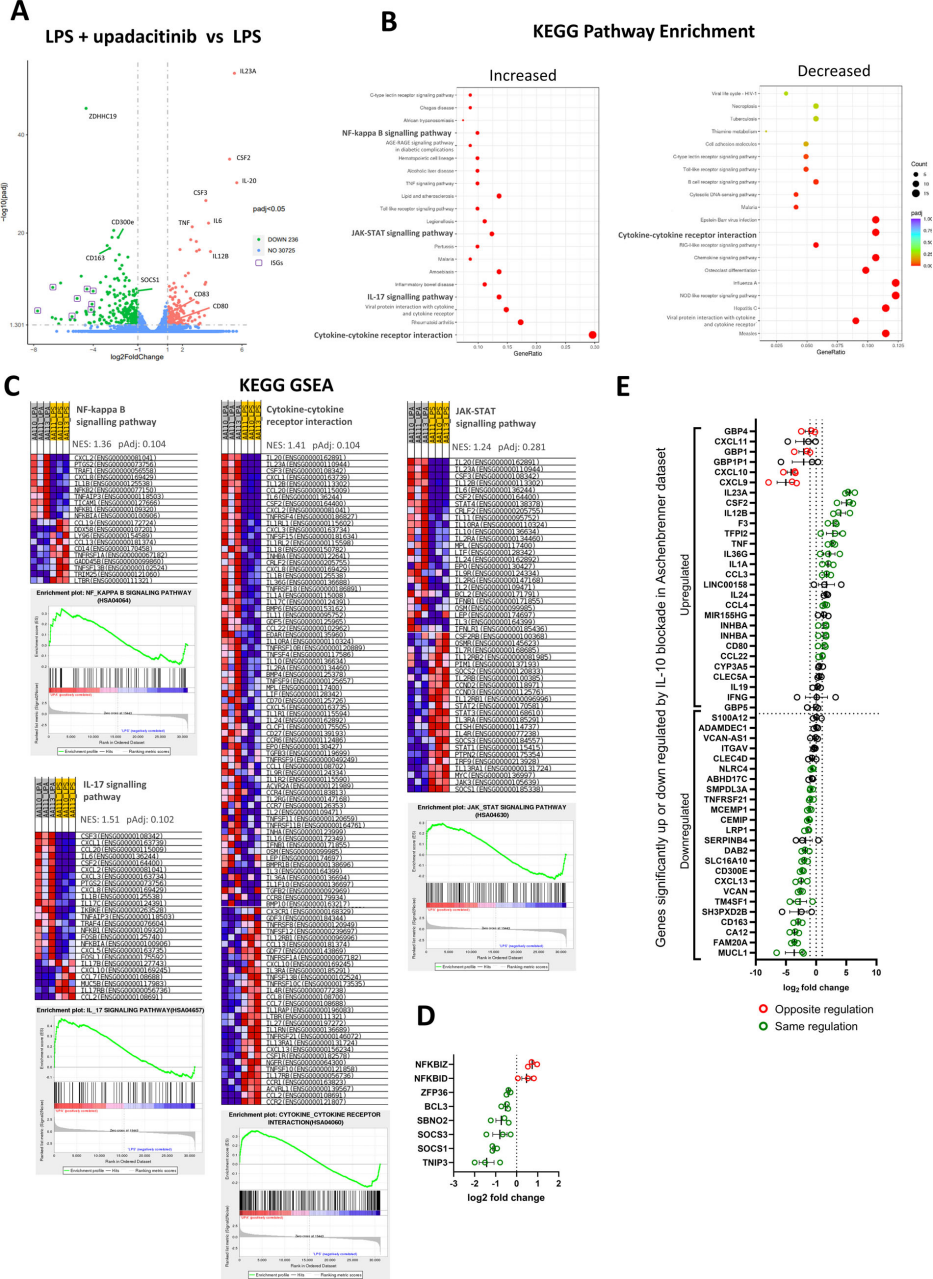
RNA sequencing (RNAseq) highlights extensive innate immune activity and indicates disrupted negative feedback. (A) Volcano plot displaying differentially expressed genes from RNAseq data between entheseal cells treated with LPS (10 ng/mL) and upadacitinib (Upa) (1 μ*M*) and entheseal cells treated with LPS alone for 12 hours. Genes of interest and interferon stimulated genes are annotated. Green indicates down‐regulated in LPS + Upa versus LPS alone. Red indicates up‐regulated in LPS + Upa versus LPS alone. (B) Dot plots of 20 most significantly up‐regulated and down‐regulated KEGG pathways enriched in LPS + Upa condition vs LPS alone. Dot size indicates counts of genes enriched in the pathway; color indicates significance. Pathways of interest are emboldened. (C) GSEA of KEGG pathways of interest highlighted in B showing normalized enrichment scores, expression heatmaps for leading edge genes in the gene set, and GSEA plots. (D) Log2 fold‐change in genes known to regulate NF‐κB activity. Green indicates down‐regulated in LPS + Upa versus LPS alone. Red indicates up‐regulated in LPS + Upa versus LPS alone. (E) Comparison of Log2 fold‐change in gene expression of our LPS + Upa versus LPS alone data set to a gene set of significantly regulated genes identified by Aschenbrenner et al^36^ comparing LPS + IL‐10R blockade to LPS alone. Colored points signify genes are significantly differentially expressed (adjusted *P* < 0.05). Green points indicate genes are regulated in the same direction in both our data set and the data set from Aschenbrenner et al^36^; red points indicate genes are regulated in the opposite direction in each data set. Data are representative of three independent experiments. Adjusted *P* values calculated using Benjamin and Hochberg's approach. GSEA, gene set enrichment analysis; IL‐10R, interleukin 10R; KEGG, Kyoto Encyclopedia of Genes and Genomes; LPS, lipopolysaccharide; *NFKBIZ*, NF‐κB inhibitor zeta; *SBNO2*, Strawberry notch homolog 2; *SOCS1*, suppressor of cytokine signaling 1; Upa, upadacitinib; *ZFP36*, zinc‐finger protein 36. Color figure can be viewed in the online issue, which is available at http://onlinelibrary.wiley.com/doi/10.1002/art.43210/abstract.

Given that JAKs are not directly involved in pathogen recognition receptor or IL‐1 family receptor signaling pathways, it seems likely that upadacitinib is impacting JAK‐dependent secondary responses downstream of myeloid differentiation factor 88 (MyD88) or NF‐κB signaling. We therefore leveraged bulk RNA sequencing (RNAseq) to observe transcriptional changes between untreated, LPS‐treated, and LPS with upadacitinib‐treated entheseal cells to determine how JAKi increased inflammatory myeloid responses. Isolated entheseal leukocytes were treated with LPS with or without 1 μ*M* of upadacitinib for 12 hours to allow for secondary response to cytokines downstream of MyD88/NF‐κB signaling before RNA isolation. Bulk RNAseq analysis highlighted increased expression of numerous proinflammatory cytokines following upadacitinib and LPS treatment (Figure 3A), confirming findings observed in Figure 2. *IL23A* (encoding IL‐23 p19) was the most significantly up‐regulated gene identified with a 10.5‐fold increase. Other key genes involved in enthesitis were up‐regulated including *CSF2* (encoding granulocyte–macrophage CSF), *IL12B*, *IL6*, and notably *CD80* and *CD83* encoding the M1‐associated costimulatory receptors. Numerous IFN‐stimulated genes (ISGs) were strongly down‐regulated by the addition of upadacitinib (Figure 3A).

KEGG pathway enrichment analysis of upadacitinib‐treated samples showed the 20 most significantly enriched pathways (Figure 3B) contained NF‐κB, JAK‐STAT, and IL‐17 signaling pathways in addition to cytokine‐cytokine receptor interaction (Figure 3B). GSEA gave normalized enrichment scores of 1.36 (adjusted *P* = 0.104), 1.24 (adjusted *P* = 0.281), 1.51 (adjusted *P* = 0.102), and 1.41 (adjusted *P* = 0.104), respectively, for each pathway (Figure 3C). Transcript levels for NF‐κB core enriched genes signaling pathways showed increased expression of inflammatory mediators and NF‐κB signaling components following upadacitinib and LPS treatment, suggesting JAK1/JAK2 inhibition may result in loss of negative regulation of NF‐κB signaling (Figure 3C).

The role of NF‐κB in driving the upadacitinib‐induced increase in IL‐23 was investigated using both THP‐1 monocytic cell line (n = 3) and peripheral blood leukocytes (n = 6). Although THP‐1 cells did not show as strong of an LPS plus upadacitinib‐dependent effect as primary cells, increased TNFα secretion when pretreated with upadacitinib was evident (Supplementary Figure S2A). An increase in NF‐κB activity via an NF‐κB–driven luciferase reporter with upadacitinib was noted (Supplementary Figure S2C). Furthermore, NF‐κB inhibition in peripheral blood leukocytes treated with a specific NF‐κB inhibitor caffeic acid phenethyl ester three hours after LPS stimulation significantly reduced secretion of IL‐23 in cells pretreated with upadacitinib (Supplementary Figure S3).

The anti‐inflammatory cytokine IL‐10 is a major regulator of NF‐κB signaling via JAK1/Tyk2 signaling, and previous reports have shown JAK1 inhibition of IL‐10 signaling may increase peripheral blood myeloid cell activity.^35^ Examination of our RNAseq data showed many IL‐10–induced regulators of NF‐κB activity were down‐regulated in the LPS with upadacitinib condition including suppressor of cytokine signaling 1 (SOCS1), SOCS3, strawberry notch homolog 2, and TNFAIP3 interacting protein 3 (TNIP3) (Figure 3D).

A recent transcriptomics report by Aschenbrenner et al^36^ identified IL‐10 as a key IL‐23 regulator in the gut. The authors treated monocytes with LPS with or without an IL‐10R–blocking antibody and used transcriptomics to identify key IL‐10 regulated genes. Examining the expression IL‐10–regulated genes in our data set compared to the data set from Aschenbrenner et al^36^ revealed a similar expression patterns with a few notable exceptions (Figure 3E). Of the 52 genes identified as significantly regulated by IL‐10 blockade in the data set from Aschenbrenner et al,^36^ 30 genes were regulated in the same direction by upadacitinib in our data set, whereas only 4 genes were oppositely regulated, suggesting a considerable amount of overlap in data sets of LPS with IL‐10 blockade and LPS with upadacitinib (Figure 3E). Those genes regulated in the opposite direction as in the data set from Aschenbrenner et al^36^ (*GBP1*, *GBP4* [encoding guanylate binding protein 1 and 4 respectively], *CXCL9*, and *CXCL10*) are known ISGs and would therefore be expected to be down‐regulated by JAK1 inhibition.

### Interrupted IL‐10 signaling as a primary driver of JAKi‐induced entheseal myeloid cell activation

**Figure 4:**
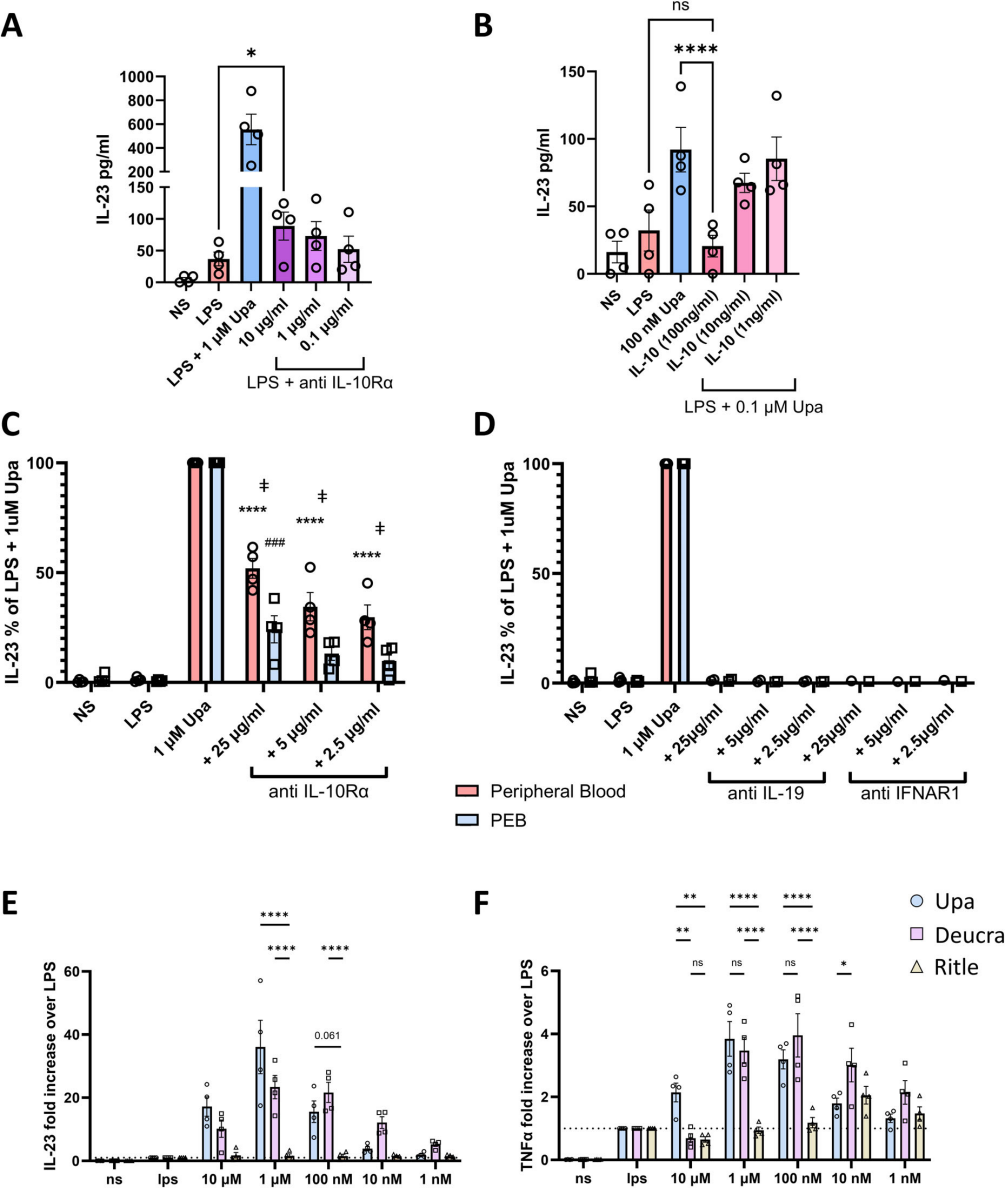
Disrupted IL‐10 and JAK1/Tyk2 negative feedback pathways lead to increased myeloid activity in JAK‐inhibited entheseal cells. (A) Entheseal cells were untreated or stimulated with LPS (10 ng/mL) and either Upa or anti–IL‐10Rα neutralizing antibody at the indicated concentrations, and secreted IL‐23 measured by enzyme‐linked immunosorbent assay (ELISA). (B) Entheseal cells treated with both LPS (10 ng/mL) and Upa (0.1 μ*M*) were given an increasing concentration of recombinant IL‐10, and secreted IL‐23 measured by ELISA. (C and D) Donor‐matched peripheral blood leukocytes (red) and entheseal cells (PEB; blue) were pretreated with indicated concentration of cytokine‐ or receptor‐neutralizing antibodies before stimulation with LPS (10 ng/mL). Data show IL‐23 secretion expressed as a percentage of IL‐23 induced by 10 ng/mL LPS + 1 μ*M* Upa. The asterisk indicates significance compared to LPS condition in peripheral blood, whereas the pound sign (#) indicates significance compared to LPS in PEB. The double dagger sign (‡) indicates significance < 0.05 between peripheral blood and PEB of the same condition. (D) Data illustrating lack of IL‐23 induction following blockade of IL‐19 or type I IFN signalling prior to LPS stimulation. (E and F) Entheseal cells were treated with LPS (10 ng/mL) with or without 10 μ*M* to 1 n*M* Upa, Deucra (Tyk2 inhibitor), or Ritle (JAK3 inhibitor) for 24 hours. (E) IL‐23 and (F) TNFα were measured by ELISA. Data are expressed as fold increase over LPS‐stimulated cells. Figures are representative of four independent experiments. *P < 0.05, **P < 0.01, ****P < 0.0001, ###*P* < 0.001, data are mean with SEM. Deucra, deucravacitinib; IL‐10, interleukin 10; LPS, lipopolysaccharide; NS, untreated entheseal cells; PEB, peri‐entheseal bone; Ritle, ritlecitinib; TNFα, tumor necrosis factor α; Upa, upadacitinib. Color figure can be viewed in the online issue, which is available at http://onlinelibrary.wiley.com/doi/10.1002/art.43210/abstract.

To assess the role of upadacitinib‐induced IL‐10 blockade in inducing increased myeloid activity, we treated entheseal cells with an IL‐10R–neutralizing antibody before stimulation with LPS and compared secretion of IL‐23 to that induced by pretreatment with upadacitinib. The addition of an IL‐10R–neutralizing antibody increased secretion of IL‐23 in a dose‐dependent manner (Figure 4A). Conversely, addition of increasing concentrations of recombinant human IL‐10 (1–100 ng/mL) to LPS‐stimulated cells pretreated with a low dose of upadacitinib (10 n*M*) reduced secretion of IL‐23 (Figure 4B). Intriguingly, blockade of IL‐10 signaling did not replicate the same levels of upadacitinib‐induced IL‐23 and showed a larger impact in peripheral blood cells than in entheseal cells. A total of 25 μg/mL of IL‐10Rα blocking antibody increased IL‐23 secretion to 50% of that induced by upadacitinib addition in peripheral blood leukocytes, yet only achieved 25% of upadacitinib‐induced IL‐23 in donor‐matched PEB leukocytes, suggesting other mechanisms play a role in the observed upadacitinib‐induced inflammation (Figure 4C).

Although RNAseq data implicated IL‐19 signaling as an up‐regulated anti‐inflammatory pathway following LPS stimulation (Supplementary Figure S4), blockade of IL‐19 did not impact IL‐23 levels (Figure 4D). Similarly, RNAseq data highlighted a down‐regulation of ISGs known to regulate MyD88 and NF‐κB driven pathways following upadacitinib addition; however, type I IFN receptor blockade did not impact IL‐23 production (Figure 4D).

### JAK1/Tyk2 pathway importance in driving JAKi‐induced myeloid inflammation

These results suggest that increased IL‐23 and other proinflammatory mediator secretion following addition of upadacitinib to LPS‐stimulated cells is largely dependent on IL‐10 signaling, and therefore JAK1/Tyk2 pathways. Therefore, we tested whether the upadacitinib‐induced increase in IL‐23 can be replicated by the use of a Tyk2 inhibitor and avoided selective JAK3 inhibition. PEB isolated cells were pretreated with pharmacologically relevant concentrations of either deucravacitinib (Tyk2 inhibitor) or ritlecitinib (JAK3 inhibitor) before stimulation with LPS. Addition of deucravacitinib to LPS‐treated PEB cells strongly induced IL‐23 (Figure 4E) and TNFα (Figure 4F) secretion in analogy to upadacitinib‐treated cells. In contrast, ritlecitinib had no significant impact on either IL‐23 or TNFα secretion, indicating the disrupted negative feedback loop is JAK1/Tyk2‐dependent further implicating IL‐10 signaling (Figure 4E and F).

### Upadacitinib promoting M1 development in LPS‐activated entheseal monocytes

**Figure 5:**
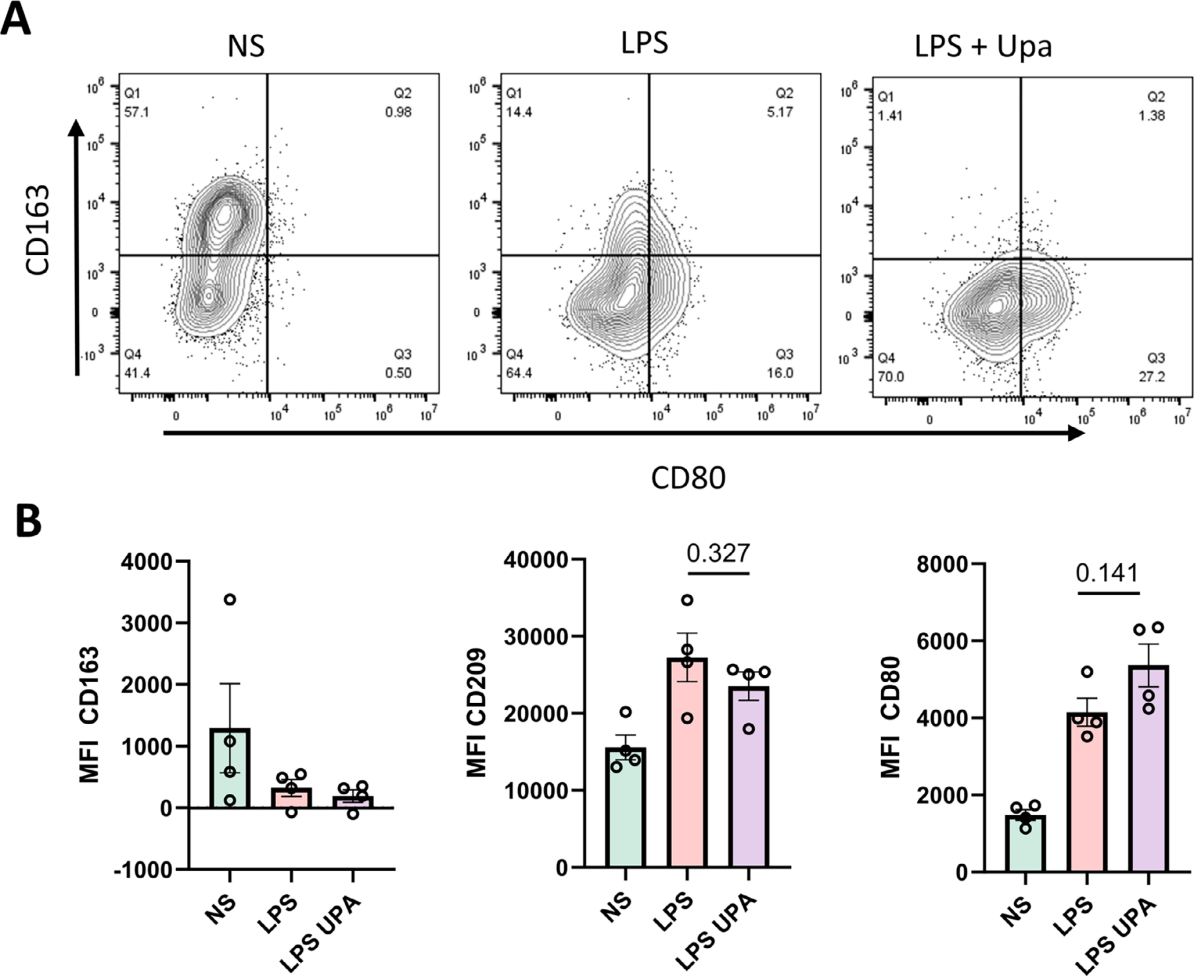
JAK1 inhibition favors M1 phenotype development in entheseal monocytes. (A) Expression of CD163, CD80, and CD209 in entheseal CD14^+^ monocytes (CD45^+^CD3^−^CD19^−^CD14^+^HLA‐DR^+^). Entheseal cells were stimulated with LPS (10 ng/mL) with or without Upa (1 μ*M*) for 24 hours and expression of M1‐phenotype marker (CD80) and M2‐phenotype markers (CD163 and CD209) were assessed by (A and B) flow cytometry. Gating strategy is shown in Supplementary Figure S1. (B) MFI was calculated for quantification of expression levels. *P* values are displayed on figures. Figures are representative of four independent experiments. Data are presented as means with SEMs. LPS, lipopolysaccharide; MFI, median fluorescence intensity; NS, untreated entheseal cells; Upa, upadacitinib. Color figure can be viewed in the online issue, which is available at http://onlinelibrary.wiley.com/doi/10.1002/art.43210/abstract.

In addition to highlighting the importance of JAK1/TYK2 signaling in controlling IL‐23 production, our RNAseq data showed the upadacitinib with LPS group had reduced expression of several M2‐associated genes compared to the LPS‐treated group. Previous work has also suggested JAKi can promote M1 development in peripheral blood mononuclear cells.^37^ We therefore explored whether upadacitinib treatment favored the development of entheseal M1 phenotype monocytes. LPS‐stimulated PEB cells pretreated with upadacitinib for 24 hours were cytometrically analyzed for monocyte CD163, CD209, and CD80 expression. Reduced CD163 and CD209 expressions and increased CD80 expression were noted compared to LPS stimulation alone (Figure 5A and B), indicating that upadacitinib promoted M1 phenotype.

### JAK mediating T cell inhibition retained in face of JAK‐mediated increased myeloid activity

Our results indicated upadacitinib activated and promoted the production of critical SpA‐driving cytokines from entheseal myeloid cells. Furthermore, KEGG pathway enrichment analysis of upadacitinib‐treated, LPS‐stimulated cells highlighted the IL‐17 signaling pathway as one of the most significantly enriched pathways (Figure 3B and C), suggesting an increased potential to drive pathologic T cell responses. These observations, however, are at odds with the clinical observation and aforementioned findings (Figure 1) that upadacitinib is an effective therapeutic in enthesitis and SpA disease. Therefore, we tested whether upadacitinib remains an effective inhibitor of T cell activity in the context of LPS and JAKi‐induced increased myeloid activity.

**Figure 6:**
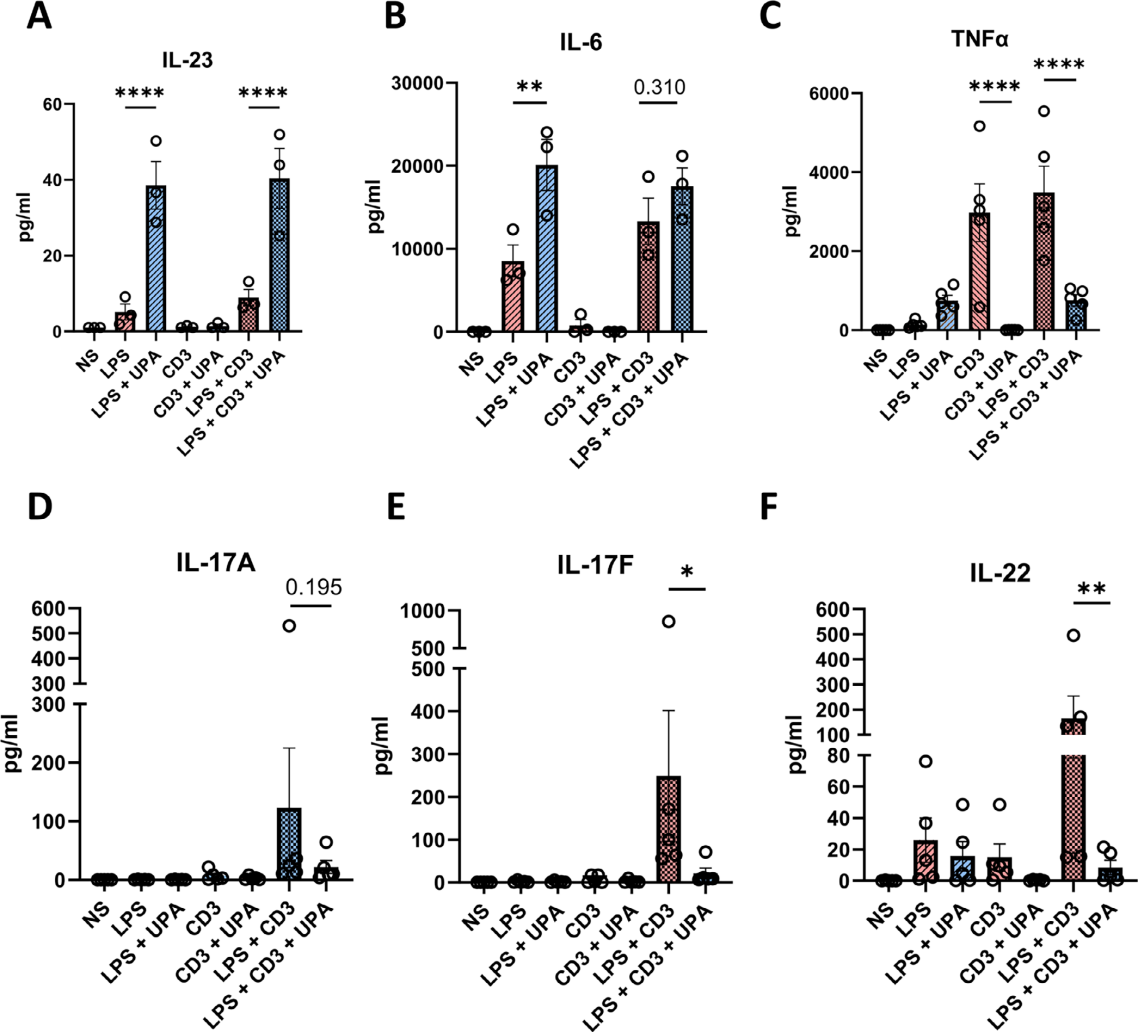
JAK1 inhibition blocks type‐17 cytokine production despite increased myeloid‐derived Th17‐driving cytokines. Entheseal cells treated with LPS (10 ng/mL) with or without Upa (1 μ*M*), anti‐CD3 (CD3) with or without upadacitinib, or LPS + anti‐CD3 with and without Upa for 48 hours. Secreted (A) IL‐23, (B) IL‐6, (C) TNFα, (D) IL‐17A, (E) IL‐17F, and (F) IL‐22 were measured by bead‐based immunoassays. Figures are representative of three (A and B) and five (C–F) independent experiments. *P < 0.05, **P < 0.01, **P < 0.001, ****P < 0.0001. Data are presented as means with SEMs. IL‐23, interleukin 23; LPS, lipopolysaccharide; NS, untreated entheseal cells; TNFα, tumor necrosis factor α; Upa, upadacitinib. Color figure can be viewed in the online issue, which is available at http://onlinelibrary.wiley.com/doi/10.1002/art.43210/abstract.

Isolated entheseal cells were stimulated with both LPS and anti‐CD3 for 48 hours to activate both myeloid and lymphoid compartments, with or without upadacitinib pretreatment. Anti‐CD28 was not used to capture the effect of the myeloid‐dependent costimulatory signal. Supernatants were collected and measured for IL‐23, IL‐6, TNFα, IL‐17A, IL‐17F, and IL‐22. Despite the observation that the addition of upadacitinib, again, increased Th17‐driving cytokines and proinflammatory cytokines when stimulated with LPS, T cell–derived IL‐17A, IL‐17F, and IL‐22 induced by LPS with anti‐CD3 remained significantly down‐regulated when pretreated with upadacitinib (Figure 6A–F) indicating that upadacitinib effectively prevented type‐17 T cell activation in the face of LPS‐mediated increased myeloid entheseal immune cells activation.

## DISCUSSION

Several cytokine‐targeted therapies have shown variable efficacy in AS and other SpA domains.^38^, ^39^ For example, despite efficacy in axial and peripheral SpA, anti–IL‐17A therapy is not effective in IBD.^40^ Another example is anti–IL‐23 therapy, which is very effective in treating psoriasis and peripheral arthritis but was inefficacious in AS.^41^ Although JAKi blocks a multitude of cytokines either directly or indirectly, a Tyk2 inhibitor that antagonizes the IL‐23 pathway has failed in UC.^21^ In the present work, we offer a molecular explanation for why JAKi may not be a panacea for SpA spectrum disorders, namely in innate immune‐driven inflammation that typically occurs in the intestine in SpA and IBD.

Although IL‐10 antagonism is not an issue for skeletal immunobiology, it may be very important for intestinal immunobiology because humans with loss‐of‐function mutations in IL‐10 or the IL‐10R are prone to early onset IBD,^22^ and IL‐10 single‐nucleotide polymorphisms are also linked to IBD.^42^ Blockade of IL‐10 by the Tyk2 class of drugs may thus not be beneficial in the intestinal environment, whereas the broader immune inhibition afforded by upadacitinib may protect against this effect in vivo in humans, perhaps via an effect on the common γ chain cytokine receptor that strongly regulates both innate and adaptive T cells.^43^ Our findings suggest that SpA spectrum disorders that are immunologically heterogeneous with predominant innate immune involvement may respond less well to JAKi therapy. In that regard, CD is strongly linked to innate immunity and barrier dysfunction via the nucleotide‐binding oligomerization domain–containing protein 2 (NOD2) pattern recognition receptor (primarily expressed by monocytes) along with other genes associated with innate immune‐driven inflammation.^44^, ^45^ Furthermore, patients with IBD have weaker major histocompatibility complex class I (MHC‐I) associations compared to the SpA spectrum disorders, but the magnitude of MHC‐I associations is greater in UC than in CD.^46^, ^47^ The efficacy of drugs that impact T cell function, such as cyclosporine and vedolizumab, is much greater in UC than CD, implying stronger population level T cell dysregulation in UC.^48^ In the light of our findings on LPS and JAKi‐mediated activation of myeloid cells, it is possible the lack of robust and consistent JAKi efficacy in CD may represent the failure to suppress a more innate immune‐centered pathology.

The enthesis is the key target tissue in SpA and PsA and the normal enthesis has a resident immune cell population in both the soft tissue immediately adjacent to the fibrocartilage and the anchoring bone. Emergent translational immunology suggests that pan‐JAKi, JAK1 inhibition, or Tyk2 inhibition works for SpA‐related arthropathy, and our current findings suggest that JAKi blocked entheseal lymphocytes key proinflammatory cytokines including TNFα, IL‐17A, and IL‐17F in keeping with the known robust efficacy of JAKi in SpA‐related arthropathy.^4^, ^8^ However, conversely, entheseal myeloid cell activation increased in LPS‐stimulated macrophage populations treated with JAKi. Yet, despite the increased activation, JAK‐mediated lymphocyte function suppression was unaffected. Given the importance of the IL‐23 pathway in the SpA‐related diseases with its abundant entheseal and intestinal myeloid cell production, our findings suggest the efficacy of JAKi in SpA is mediated via the adaptive immune system in the face of potential increased activity within the myeloid compartment. This appears to be clinically relevant in the context of Tyk2 inhibition that has failed in UC (thus far reported in a press release).^21^

Our findings at first glance seem counterintuitive, in other words, why would the elevation of several myeloid‐related cytokines be associated with good arthritis disease control? As shown in the summary schematic diagram of the mechanisms at play (see the graphical abstract), we traced such cytokine elevations to entheseal myeloid cell IL‐10 pathway inhibition and subsequent failure to initiate negative feedback to control drivers of proinflammatory cytokine production such as NF‐κB. Indeed, IL‐10–specific blockade emulated the results seen in upadacitinib‐treated cells and specific inhibition of NF‐κB three hours after stimulation with LPS in peripheral blood leukocytes that had been pretreated with upadacitinib significantly reduced the secretion of IL‐23 (Supplementary Figure S3). The importance of paracrine IL‐10 in controlling gut monocyte IL‐23 expression has been reported previously by Aschenbrenner et al,^36^ and the transcriptional changes measured in cells treated with LPS and anti–IL‐10Rα antibody closely resembles that of entheseal cells treated with LPS and upadacitinib in our work (Figure 3C). Although they did not elucidate the mechanisms by which IL‐10 induced the negative feedback, we noted a decrease in the expression of several IL‐10‐regulated genes known to regulate NF‐κB activity, most notably SOCS1 and SOCS3 (Figure 3D); however, further work is required to understand their impact on entheseal monocyte‐derived IL‐23.

Previous studies have shown that blood myeloid cell JAKi was associated with increased cytokine production.^35^, ^37^, ^49^ Although strongly IL‐10–dependent, blockade of IL‐10 did not completely replicate the increased levels of cytokines observed by upadacitinib addition. This may, in part, be due to the high receptor density of IL‐10R on human monocytes, making complete inhibition of signaling using receptor‐specific antibodies challenging.^50^ This difference was particularly notable in the entheseal leukocytes compared to peripheral blood, in which IL‐23 was only up‐regulated to 25% of upadacitinib‐induced levels versus 50% in blood by blockade of IL‐10 (Figure 4C). The failure of JAK3 inhibition to replicate the observed increases in IL‐23 and TNFα following JAK1 and Tyk2 inhibition strongly implicates JAK1/Tyk2 pathways in this mechanism, yet blockade of IFN signaling and IL‐19 (both implicated in transcriptomic data) did not impact cytokine production (Figure 4D). These results suggest, in addition to IL‐10, other unknown mechanisms are also contributing to the observed increase in proinflammatory cytokines and may be more important in entheseal biology than in peripheral blood.

One of the study's limitations is that upadacitinib also has efficacy against JAK2 signaling. We were unable to rule out JAK2 involvement because of the lack of highly specific JAK2 inhibitors, therefore the observed effect of using upadacitinib and deucravacitinib may not only be due to a JAK1/Tyk2‐dependent manner but also may involve JAK2 signaling. Although previous work has suggested JAK3 inhibition to promote myeloid production of proinflammatory cytokines,^49^ these experiments were performed with low‐dose tofacitinib, which is now known not to be JAK3 specific. Furthermore, in our hands, addition of the highly selective JAK3 inhibitor ritlecitinib to LPS‐stimulated entheseal cells did not promote secretion of SpA‐driving cytokines, namely IL‐23 or TNFα. Interestingly, TNFα, which is produced by both myeloid and T cells, was up‐regulated by addition of upadacitinib to LPS‐treated cells, yet only to approximately 25% of the output induced from T cells by TCR activation alone. Furthermore, as previously observed, the addition of upadacitinib to TCR‐activated T cells completely ablated TNFα production. Yet, when cells are stimulated with both LPS and anti‐CD3 and pretreated with upadacitinib, TNFα can only be reduced to the levels observed in LPS‐ and upadacitinib‐treated myeloid cells, indicating that upadacitinib may independently increase myeloid cells TNFα production while also inhibiting T cell TNFα production, with these findings pointing to a key role of conventional T cells in vivo in SpA pathogenesis rather than TNFα from other sources.

In conclusion, JAKi in an in vitro enthesitis model system was associated with disparate impacts of the myeloid and lymphoid compartments and suggested that although upadacitinib effectively blocked the adaptive immune system, LPS‐mediated innate immunity was preserved or even up‐regulated following JAKi. Given the pervasive gut barrier dysfunction in SpA, bacterial molecules including LPS seem clinically relevant. Given the immunologic heterogeneity in SpA spectrum disorders and allied conditions, these findings provide a map toward therapy in SpA spectrum disease and why JAKi, especially with Tyk2 blockade in IBD, may not be a SpA disease spectrum panacea. In essence, the blockade of IL‐10 by JAKi, especially Tyk2 inhibition, may lead to a differential effect of therapy across SpA spectrum joint and intestinal disease. One limitation of our work is the lack of data from IBD tissues, but the work from Aschenbrenner et al^36^ strongly demonstrates the importance of IL‐10 in the control of IL‐23 in gut monocytes, which suggests identical mechanism are also operational in IBD. Additionally, genome‐wide association studies clearly show the importance of innate immune dysregulation in CD.^45^ Our findings offer an explanation as to why Tyk2 antagonism and potential detrimental impacts on IL‐10 signaling may account for divergent therapy landscape between intestinal and skeletal immunotherapy under the more restricted cytokine antagonism via Tyk2 blockade compared to other JAKs.

## AUTHOR CONTRIBUTIONS

All authors contributed to at least one of the following manuscript preparation roles: conceptualization AND/OR methodology, software, investigation, formal analysis, data curation, visualization, and validation AND drafting or reviewing/editing the final draft. As corresponding author, Dr McGonagle confirms that all authors have provided the final approval of the version to be published and takes responsibility for the affirmations regarding article submission (eg, not under consideration by another journal), the integrity of the data presented, and the statements regarding compliance with institutional review board/Declaration of Helsinki requirements.

## ROLE OF THE STUDY SPONSOR

AbbVie Inc had no role in the study design or in the collection, analysis, or interpretation of the data, the writing of the manuscript, or the decision to submit the manuscript for publication. Publication of this article was not contingent upon approval by AbbVie Inc.

## Supporting information


**Disclosure form**.

**Appendix S1:** Supplementary Information
