# Supplementary material for: Paradoxical Activation of Entheseal Myeloid Cells by JAK1 and Tyk2 Inhibitors via Interleukin‐10 Antagonism
Source: Arthritis Rheumatol. 2025 Jul 4;77(10):1373–86. doi: 10.1002/art.43210 (PMC12479184; doi:10.1002/art.43210)
Supplement: Supplementary file 1 — Disclosure form. [file ART-77-1373-s002.pdf]

ICMJE DISCLOSURE FORM

Date: 31/03/2025

Your Name: Chi Wong

Manuscript Title **Paradoxical Activation of Entheseal Myeloid Cells by JAK1 and TYK2 Inhibitors via IL10 Antagonism**
Manuscript number (if known):

In the interest of transparency, we ask you to disclose all relationships/activities/interests listed below that are related to the content of your manuscript. "Related" means any relation with for-profit or not-for-profit third parties whose interests may be affected by the content of the manuscript. Disclosure represents a commitment to transparency and does not necessarily indicate a bias. If you are in doubt about whether to list a relationship/activity/interest, it is preferable that you do so.

The following questions apply to the author's relationships/activities/interests as they relate to the current manuscript only.

The author's relationships/activities/interests should be defined broadly. For example, if your manuscript pertains to the epidemiology of hypertension, you should declare all relationships with manufacturers of antihypertensive medication, even if that medication is not mentioned in the manuscript.

In item #1 below, report all support for the work reported in this manuscript without time limit. For all other items, the time frame for disclosure is the past 36 months.

		Name all entities with whom you have this relationship or indicate none (add rows as needed)	Specifications/Comments (e.g., if payments were made to you or to your institution)
		**Time frame: Since the initial planning of the work**	
1	All support for the present manuscript (e.g., funding, provision of study materials, medical writing, article processing charges, etc.) **No time limit for this item.**	X____None	
		**Time frame: past 36 months**	
2	Grants or contracts from any entity (if not indicated in item #1 above).	X____None	
3	Royalties or licenses	X____None	
4	Consulting fees	X____None	

5	Payment or honoraria for lectures, presentations, speakers bureaus, manuscript writing or educational events	X___None	
6	Payment for expert testimony	X___None	
7	Support for attending meetings and/or travel	X___None	
8	Patents planned, issued or pending	X___None	
9	Participation on a Data Safety Monitoring Board or Advisory Board	X___None	
10	Leadership or fiduciary role in other board, society, committee or advocacy group, paid or unpaid	X___None	
11	Stock or stock options	X___None	
12	Receipt of equipment, materials, drugs, medical writing, gifts or other services	X___None	
13	Other financial or non-financial interests	X___None	

Please place an "X" next to the following statement to indicate your agreement:

X ___ I certify that I have answered every question and have not altered the wording of any of the questions on this form.

ICMJE DISCLOSURE FORM

Date: 31/03/2025

Your Name: Tristan McMillan

Manuscript Title **Paradoxical Activation of Entheseal Myeloid Cells by JAK1 and TYK2 Inhibitors via IL10 Antagonism**
Manuscript number (if known):

In the interest of transparency, we ask you to disclose all relationships/activities/interests listed below that are related to the content of your manuscript. "Related" means any relation with for-profit or not-for-profit third parties whose interests may be affected by the content of the manuscript. Disclosure represents a commitment to transparency and does not necessarily indicate a bias. If you are in doubt about whether to list a relationship/activity/interest, it is preferable that you do so.

The following questions apply to the author's relationships/activities/interests as they relate to the current manuscript only.

The author's relationships/activities/interests should be defined broadly. For example, if your manuscript pertains to the epidemiology of hypertension, you should declare all relationships with manufacturers of antihypertensive medication, even if that medication is not mentioned in the manuscript.

In item #1 below, report all support for the work reported in this manuscript without time limit. For all other items, the time frame for disclosure is the past 36 months.

		Name all entities with whom you have this relationship or indicate none (add rows as needed)	Specifications/Comments (e.g., if payments were made to you or to your institution)
		**Time frame: Since the initial planning of the work**	
1	All support for the present manuscript (e.g., funding, provision of study materials, medical writing, article processing charges, etc.) **No time limit for this item.**	X None	
		**Time frame: past 36 months**	
2	Grants or contracts from any entity (if not indicated in item #1 above).	X None	
3	Royalties or licenses	X None	
4	Consulting fees	X None	

5	Payment or honoraria for lectures, presentations, speakers bureaus, manuscript writing or educational events	X___None	
6	Payment for expert testimony	X___None	
7	Support for attending meetings and/or travel	X___None	
8	Patents planned, issued or pending	X___None	
9	Participation on a Data Safety Monitoring Board or Advisory Board	X___None	
10	Leadership or fiduciary role in other board, society, committee or advocacy group, paid or unpaid	X___None	
11	Stock or stock options	X___None	
12	Receipt of equipment, materials, drugs, medical writing, gifts or other services	X___None	
13	Other financial or non-financial interests	X___None	

Please place an "X" next to the following statement to indicate your agreement:

__X_ I certify that I have answered every question and have not altered the wording of any of the questions on this form.

ICMJE DISCLOSURE FORM

Date: 31/03/2025

Your Name: Kerem Abacar
Manuscript Title **Paradoxical Activation of Entheseal Myeloid Cells by JAK1 and TYK2 Inhibitors via IL10 Antagonism**
Manuscript number (if known):

In the interest of transparency, we ask you to disclose all relationships/activities/interests listed below that are related to the content of your manuscript. "Related" means any relation with for-profit or not-for-profit third parties whose interests may be affected by the content of the manuscript. Disclosure represents a commitment to transparency and does not necessarily indicate a bias. If you are in doubt about whether to list a relationship/activity/interest, it is preferable that you do so.

The following questions apply to the author's relationships/activities/interests as they relate to the current manuscript only.

The author's relationships/activities/interests should be defined broadly. For example, if your manuscript pertains to the epidemiology of hypertension, you should declare all relationships with manufacturers of antihypertensive medication, even if that medication is not mentioned in the manuscript.

In item #1 below, report all support for the work reported in this manuscript without time limit. For all other items, the time frame for disclosure is the past 36 months.

		Name all entities with whom you have this relationship or indicate none (add rows as needed)	Specifications/Comments (e.g., if payments were made to you or to your institution)
		Time frame: Since the initial planning of the work	
1	All support for the present manuscript (e.g., funding, provision of study materials, medical writing, article processing charges, etc.) **No time limit for this item.**	_X___None	
		Time frame: past 36 months	
2	Grants or contracts from any entity (if not indicated in item #1 above).	____ PARTNER Fellowship programı (Amgen)	
3	Royalties or licenses	_X___None	
4	Consulting fees	_X___None	

5	Payment or honoraria for lectures, presentations, speakers bureaus, manuscript writing or educational events	X___None	
6	Payment for expert testimony	X___None	
7	Support for attending meetings and/or travel	X___None	
8	Patents planned, issued or pending	X___None	
9	Participation on a Data Safety Monitoring Board or Advisory Board	X___None	
10	Leadership or fiduciary role in other board, society, committee or advocacy group, paid or unpaid	X___None	
11	Stock or stock options	X___None	
12	Receipt of equipment, materials, drugs, medical writing, gifts or other services	X___None	
13	Other financial or non-financial interests	X___None	

Please place an "X" next to the following statement to indicate your agreement:

__X_ I certify that I have answered every question and have not altered the wording of any of the questions on this form.

ICMJE DISCLOSURE FORM

Date: 31/03/2025

Your Name: Charlie Bridgewood

Manuscript Title **Paradoxical Activation of Entheseal Myeloid Cells by JAK1 and TYK2 Inhibitors via IL10 Antagonism**
Manuscript number (if known):

In the interest of transparency, we ask you to disclose all relationships/activities/interests listed below that are related to the content of your manuscript. "Related" means any relation with for-profit or not-for-profit third parties whose interests may be affected by the content of the manuscript. Disclosure represents a commitment to transparency and does not necessarily indicate a bias. If you are in doubt about whether to list a relationship/activity/interest, it is preferable that you do so.

The following questions apply to the author's relationships/activities/interests as they relate to the current manuscript only.

The author's relationships/activities/interests should be defined broadly. For example, if your manuscript pertains to the epidemiology of hypertension, you should declare all relationships with manufacturers of antihypertensive medication, even if that medication is not mentioned in the manuscript.

In item #1 below, report all support for the work reported in this manuscript without time limit. For all other items, the time frame for disclosure is the past 36 months.

		Name all entities with whom you have this relationship or indicate none (add rows as needed)	Specifications/Comments (e.g., if payments were made to you or to your institution)
	**Time frame: Since the initial planning of the work**		
1	All support for the present manuscript (e.g., funding, provision of study materials, medical writing, article processing charges, etc.) **No time limit for this item.**	X___None	
	**Time frame: past 36 months**		
2	Grants or contracts from any entity (if not indicated in item #1 above).	X___None	
3	Royalties or licenses	X___None	
4	Consulting fees	X___None	

5	Payment or honoraria for lectures, presentations, speakers bureaus, manuscript writing or educational events	X___None	
6	Payment for expert testimony	X___None	
7	Support for attending meetings and/or travel	X___None	
8	Patents planned, issued or pending	X___None	
9	Participation on a Data Safety Monitoring Board or Advisory Board	X___None	
10	Leadership or fiduciary role in other board, society, committee or advocacy group, paid or unpaid	X___None	
11	Stock or stock options	X___None	
12	Receipt of equipment, materials, drugs, medical writing, gifts or other services	X___None	
13	Other financial or non-financial interests	X___None	

Please place an "X" next to the following statement to indicate your agreement:

X ___ I certify that I have answered every question and have not altered the wording of any of the questions on this form.

ICMJE DISCLOSURE FORM

Date: 31/03/2025

Your Name: Nicole McDermott
Manuscript Title **Paradoxical Activation of Entheseal Myeloid Cells by JAK1 and TYK2 Inhibitors via IL10 Antagonism**
Manuscript number (if known):

In the interest of transparency, we ask you to disclose all relationships/activities/interests listed below that are related to the content of your manuscript. "Related" means any relation with for-profit or not-for-profit third parties whose interests may be affected by the content of the manuscript. Disclosure represents a commitment to transparency and does not necessarily indicate a bias. If you are in doubt about whether to list a relationship/activity/interest, it is preferable that you do so.

The following questions apply to the author's relationships/activities/interests as they relate to the current manuscript only.

The author's relationships/activities/interests should be defined broadly. For example, if your manuscript pertains to the epidemiology of hypertension, you should declare all relationships with manufacturers of antihypertensive medication, even if that medication is not mentioned in the manuscript.

In item #1 below, report all support for the work reported in this manuscript without time limit. For all other items, the time frame for disclosure is the past 36 months.

		Name all entities with whom you have this relationship or indicate none (add rows as needed)	Specifications/Comments (e.g., if payments were made to you or to your institution)
		**Time frame: Since the initial planning of the work**	
1	All support for the present manuscript (e.g., funding, provision of study materials, medical writing, article processing charges, etc.) **No time limit for this item.**	X___None	
		**Time frame: past 36 months**	
2	Grants or contracts from any entity (if not indicated in item #1 above).	X___None	
3	Royalties or licenses	X___None	
4	Consulting fees	X___None	

5	Payment or honoraria for lectures, presentations, speakers bureaus, manuscript writing or educational events	X____None	
6	Payment for expert testimony	X____None	
7	Support for attending meetings and/or travel	X____None	
8	Patents planned, issued or pending	X____None	
9	Participation on a Data Safety Monitoring Board or Advisory Board	X____None	
10	Leadership or fiduciary role in other board, society, committee or advocacy group, paid or unpaid	X____None	
11	Stock or stock options	X____None	
12	Receipt of equipment, materials, drugs, medical writing, gifts or other services	X____None	
13	Other financial or non-financial interests	X____None	

Please place an "X" next to the following statement to indicate your agreement:

_X__ I certify that I have answered every question and have not altered the wording of any of the questions on this form.

ICMJE DISCLOSURE FORM

Date: 31/03/2025

Your Name: Mark Harland

Manuscript Title **Paradoxical Activation of Entheseal Myeloid Cells by JAK1 and TYK2 Inhibitors via IL10 Antagonism**
Manuscript number (if known):

In the interest of transparency, we ask you to disclose all relationships/activities/interests listed below that are related to the content of your manuscript. "Related" means any relation with for-profit or not-for-profit third parties whose interests may be affected by the content of the manuscript. Disclosure represents a commitment to transparency and does not necessarily indicate a bias. If you are in doubt about whether to list a relationship/activity/interest, it is preferable that you do so.

The following questions apply to the author's relationships/activities/interests as they relate to the current manuscript only.

The author's relationships/activities/interests should be defined broadly. For example, if your manuscript pertains to the epidemiology of hypertension, you should declare all relationships with manufacturers of antihypertensive medication, even if that medication is not mentioned in the manuscript.

In item #1 below, report all support for the work reported in this manuscript without time limit. For all other items, the time frame for disclosure is the past 36 months.

		Name all entities with whom you have this relationship or indicate none (add rows as needed)	Specifications/Comments (e.g., if payments were made to you or to your institution)
		Time frame: Since the initial planning of the work	
1	All support for the present manuscript (e.g., funding, provision of study materials, medical writing, article processing charges, etc.) **No time limit for this item.**	X____None	
		Time frame: past 36 months	
2	Grants or contracts from any entity (if not indicated in item #1 above).	X____None	
3	Royalties or licenses	X____None	
4	Consulting fees	X____None	

5	Payment or honoraria for lectures, presentations, speakers bureaus, manuscript writing or educational events	X___None	
6	Payment for expert testimony	X___None	
7	Support for attending meetings and/or travel	X___None	
8	Patents planned, issued or pending	X___None	
9	Participation on a Data Safety Monitoring Board or Advisory Board	X___None	
10	Leadership or fiduciary role in other board, society, committee or advocacy group, paid or unpaid	X___None	
11	Stock or stock options	X___None	
12	Receipt of equipment, materials, drugs, medical writing, gifts or other services	X___None	
13	Other financial or non-financial interests	X___None	

Please place an "X" next to the following statement to indicate your agreement:

_X_ I certify that I have answered every question and have not altered the wording of any of the questions on this form.

ICMJE DISCLOSURE FORM

Date: 31/03/2025

Your Name: Tom Macleod

Manuscript Title **Paradoxical Activation of Entheseal Myeloid Cells by JAK1 and TYK2 Inhibitors via IL10 Antagonism**
Manuscript number (if known):

In the interest of transparency, we ask you to disclose all relationships/activities/interests listed below that are related to the content of your manuscript. "Related" means any relation with for-profit or not-for-profit third parties whose interests may be affected by the content of the manuscript. Disclosure represents a commitment to transparency and does not necessarily indicate a bias. If you are in doubt about whether to list a relationship/activity/interest, it is preferable that you do so.

The following questions apply to the author's relationships/activities/interests as they relate to the current manuscript only.

The author's relationships/activities/interests should be defined broadly. For example, if your manuscript pertains to the epidemiology of hypertension, you should declare all relationships with manufacturers of antihypertensive medication, even if that medication is not mentioned in the manuscript.

In item #1 below, report all support for the work reported in this manuscript without time limit. For all other items, the time frame for disclosure is the past 36 months.

		Name all entities with whom you have this relationship or indicate none (add rows as needed)	Specifications/Comments (e.g., if payments were made to you or to your institution)
**Time frame: Since the initial planning of the work**			
1	All support for the present manuscript (e.g., funding, provision of study materials, medical writing, article processing charges, etc.) **No time limit for this item.**	AbbVie Pharmaceuticals	Provided investigator initiated funding to University for project
**Time frame: past 36 months**			
2	Grants or contracts from any entity (if not indicated in item #1 above).	X None	
3	Royalties or licenses	X None	
4	Consulting fees	X None	

5	Payment or honoraria for lectures, presentations, speakers bureaus, manuscript writing or educational events	X___None	
6	Payment for expert testimony	X___None	
7	Support for attending meetings and/or travel	X___None	
8	Patents planned, issued or pending	X___None	
9	Participation on a Data Safety Monitoring Board or Advisory Board	X___None	
10	Leadership or fiduciary role in other board, society, committee or advocacy group, paid or unpaid	X___None	
11	Stock or stock options	X___None	
12	Receipt of equipment, materials, drugs, medical writing, gifts or other services	X___None	
13	Other financial or non-financial interests	X___None	

Please place an "X" next to the following statement to indicate your agreement:

_X_ I certify that I have answered every question and have not altered the wording of any of the questions on this form.

ICMJE DISCLOSURE FORM

Date: 31/03/2025

Your Name: Ala Altaie

Manuscript Title **Paradoxical Activation of Entheseal Myeloid Cells by JAK1 and TYK2 Inhibitors via IL10 Antagonism**
Manuscript number (if known):

In the interest of transparency, we ask you to disclose all relationships/activities/interests listed below that are related to the content of your manuscript. "Related" means any relation with for-profit or not-for-profit third parties whose interests may be affected by the content of the manuscript. Disclosure represents a commitment to transparency and does not necessarily indicate a bias. If you are in doubt about whether to list a relationship/activity/interest, it is preferable that you do so.

The following questions apply to the author's relationships/activities/interests as they relate to the current manuscript only.

The author's relationships/activities/interests should be defined broadly. For example, if your manuscript pertains to the epidemiology of hypertension, you should declare all relationships with manufacturers of antihypertensive medication, even if that medication is not mentioned in the manuscript.

In item #1 below, report all support for the work reported in this manuscript without time limit. For all other items, the time frame for disclosure is the past 36 months.

		Name all entities with whom you have this relationship or indicate none (add rows as needed)	Specifications/Comments (e.g., if payments were made to you or to your institution)
Time frame: Since the initial planning of the work			
1	All support for the present manuscript (e.g., funding, provision of study materials, medical writing, article processing charges, etc.) **No time limit for this item.**	X None	
Time frame: past 36 months			
2	Grants or contracts from any entity (if not indicated in item #1 above).	X None	
3	Royalties or licenses	X None	
4	Consulting fees	X None	

5	Payment or honoraria for lectures, presentations, speakers bureaus, manuscript writing or educational events	X___None	
6	Payment for expert testimony	X___None	
7	Support for attending meetings and/or travel	X___None	
8	Patents planned, issued or pending	X___None	
9	Participation on a Data Safety Monitoring Board or Advisory Board	X___None	
10	Leadership or fiduciary role in other board, society, committee or advocacy group, paid or unpaid	X___None	
11	Stock or stock options	X___None	
12	Receipt of equipment, materials, drugs, medical writing, gifts or other services	X___None	
13	Other financial or non-financial interests	X___None	

Please place an "X" next to the following statement to indicate your agreement:

X I certify that I have answered every question and have not altered the wording of any of the questions on this form.

ICMJE DISCLOSURE FORM

Date: 31/03/2025

Your Name: Dennis McGonagle

Manuscript Title **Paradoxical Activation of Entheseal Myeloid Cells by JAK1 and TYK2 Inhibitors via IL10 Antagonism**
Manuscript number (if known):

In the interest of transparency, we ask you to disclose all relationships/activities/interests listed below that are related to the content of your manuscript. “Related” means any relation with for-profit or not-for-profit third parties whose interests may be affected by the content of the manuscript. Disclosure represents a commitment to transparency and does not necessarily indicate a bias. If you are in doubt about whether to list a relationship/activity/interest, it is preferable that you do so.

The following questions apply to the author’s relationships/activities/interests as they relate to the current manuscript only.

The author’s relationships/activities/interests should be defined broadly. For example, if your manuscript pertains to the epidemiology of hypertension, you should declare all relationships with manufacturers of antihypertensive medication, even if that medication is not mentioned in the manuscript.

In item #1 below, report all support for the work reported in this manuscript without time limit. For all other items, the time frame for disclosure is the past 36 months.

		Name all entities with whom you have this relationship or indicate none (add rows as needed)	Specifications/Comments (e.g., if payments were made to you or to your institution)
		**Time frame: Since the initial planning of the work**	
1	All support for the present manuscript (e.g., funding, provision of study materials, medical writing, article processing charges, etc.) **No time limit for this item.**	____Abbvie Pharmaceuticals	Investigator initiated funding
		**Time frame: past 36 months**	
2	Grants or contracts from any entity (if not indicated in item #1 above).	X____None	
3	Royalties or licenses	X____None	

4	Consulting fees	___Speaker fees from Abbvie	
5	Payment or honoraria for lectures, presentations, speakers bureaus, manuscript writing or educational events	____Fees for lectures	
6	Payment for expert testimony	X____None	
7	Support for attending meetings and/or travel	X____None	
8	Patents planned, issued or pending	X____None	
9	Participation on a Data Safety Monitoring Board or Advisory Board	X____None	
10	Leadership or fiduciary role in other board, society, committee or advocacy group, paid or unpaid	X____None	
11	Stock or stock options	X____None	
12	Receipt of equipment, materials, drugs, medical writing, gifts or other services	X____None	
13	Other financial or non-financial interests	X____None	

Please place an "X" next to the following statement to indicate your agreement:

_X__ I certify that I have answered every question and have not altered the wording of any of the questions on this form.

ICMJE DISCLOSURE FORM

Date:31/03/2025
Your Name: Sami Giryes
Manuscript Title **Paradoxical Activation of Entheseal Myeloid Cells by JAK1 and TYK2 Inhibitors via IL10 Antagonism**
Manuscript number (if known)

In the interest of transparency, we ask you to disclose all relationships/activities/interests listed below that are related to the content of your manuscript. "Related" means any relation with for-profit or not-for-profit third parties whose interests may be affected by the content of the manuscript. Disclosure represents a commitment to transparency and does not necessarily indicate a bias. If you are in doubt about whether to list a relationship/activity/interest, it is preferable that you do so.

The following questions apply to the author's relationships/activities/interests as they relate to the current manuscript only.

The author's relationships/activities/interests should be defined broadly. For example, if your manuscript pertains to the epidemiology of hypertension, you should declare all relationships with manufacturers of antihypertensive medication, even if that medication is not mentioned in the manuscript.

In item #1 below, report all support for the work reported in this manuscript without time limit. For all other items, the time frame for disclosure is the past 36 months.

		Name all entities with whom you have this relationship or indicate none (add rows as needed)	Specifications/Comments (e.g., if payments were made to you or to your institution)
		**Time frame: Since the initial planning of the work**	
1	All support for the present manuscript (e.g., funding, provision of study materials, medical writing, article processing charges, etc.) **No time limit for this item.**	X___None	
		**Time frame: past 36 months**	
2	Grants or contracts from any entity (if not indicated in item #1 above).	PARTNER (Psoriatic Arthritis Network fellowship program), which is funded by Amgen	
3	Royalties or licenses	X___None	

4	Consulting fees	____fees for consulting AbbVie	
5	Payment or honoraria for lectures, presentations, speakers bureaus, manuscript writing or educational events	____fees for participating in educational events - AbbVie	
6	Payment for expert testimony	_X___None	
7	Support for attending meetings and/or travel	_X___None	
8	Patents planned, issued or pending	_X___None	
9	Participation on a Data Safety Monitoring Board or Advisory Board	_X___None	
10	Leadership or fiduciary role in other board, society, committee or advocacy group, paid or unpaid	_X___None	
11	Stock or stock options	_X___None	
12	Receipt of equipment, materials, drugs, medical writing, gifts or other services	_X___None	
13	Other financial or non-financial interests	_X___None	

Please place an "X" next to the following statement to indicate your agreement:

_X__ I certify that I have answered every question and have not altered the wording of any of the questions on this form.

ICMJE DISCLOSURE FORM

Date:31/03/2025
Your Name:Abhay Rao
Manuscript Title **Paradoxical Activation of Entheseal Myeloid Cells by JAK1 and TYK2 Inhibitors via IL10 Antagonism**
Manuscript number (if known):____

In the interest of transparency, we ask you to disclose all relationships/activities/interests listed below that are related to the content of your manuscript. "Related" means any relation with for-profit or not-for-profit third parties whose interests may be affected by the content of the manuscript. Disclosure represents a commitment to transparency and does not necessarily indicate a bias. If you are in doubt about whether to list a relationship/activity/interest, it is preferable that you do so.

The following questions apply to the author's relationships/activities/interests as they relate to the current manuscript only.

The author's relationships/activities/interests should be defined broadly. For example, if your manuscript pertains to the epidemiology of hypertension, you should declare all relationships with manufacturers of antihypertensive medication, even if that medication is not mentioned in the manuscript.

In item #1 below, report all support for the work reported in this manuscript without time limit. For all other items, the time frame for disclosure is the past 36 months.

		Name all entities with whom you have this relationship or indicate none (add rows as needed)	Specifications/Comments (e.g., if payments were made to you or to your institution)
Time frame: Since the initial planning of the work			
1	All support for the present manuscript (e.g., funding, provision of study materials, medical writing, article processing charges, etc.) **No time limit for this item.**	X___None	
Time frame: past 36 months			
2	Grants or contracts from any entity (if not indicated in item #1 above).	X___None	
3	Royalties or licenses	X___None	
4	Consulting fees	X___None	
5		X___None	

	Payment or honoraria for lectures, presentations, speakers bureaus, manuscript writing or educational events		
6	Payment for expert testimony	X____None	
7	Support for attending meetings and/or travel	X____None	
8	Patents planned, issued or pending	X____None	
9	Participation on a Data Safety Monitoring Board or Advisory Board	X____None	
10	Leadership or fiduciary role in other board, society, committee or advocacy group, paid or unpaid	X____None	
11	Stock or stock options	X____None	
12	Receipt of equipment, materials, drugs, medical writing, gifts or other services	X____None	
13	Other financial or non-financial interests	X____None	

Please place an "X" next to the following statement to indicate your agreement:

X_ I certify that I have answered every question and have not altered the wording of any of the questions on this form.

ICMJE DISCLOSURE FORM

Date:31/03/2025
Your Name Almas Khan
Manuscript Title **Paradoxical Activation of Entheseal Myeloid Cells by JAK1 and TYK2 Inhibitors via IL10 Antagonism**
Manuscript number (if known):____

In the interest of transparency, we ask you to disclose all relationships/activities/interests listed below that are related to the content of your manuscript. "Related" means any relation with for-profit or not-for-profit third parties whose interests may be affected by the content of the manuscript. Disclosure represents a commitment to transparency and does not necessarily indicate a bias. If you are in doubt about whether to list a relationship/activity/interest, it is preferable that you do so.

The following questions apply to the author's relationships/activities/interests as they relate to the current manuscript only.

The author's relationships/activities/interests should be defined broadly. For example, if your manuscript pertains to the epidemiology of hypertension, you should declare all relationships with manufacturers of antihypertensive medication, even if that medication is not mentioned in the manuscript.

In item #1 below, report all support for the work reported in this manuscript without time limit. For all other items, the time frame for disclosure is the past 36 months.

		Name all entities with whom you have this relationship or indicate none (add rows as needed)	Specifications/Comments (e.g., if payments were made to you or to your institution)
		**Time frame: Since the initial planning of the work**	
1	All support for the present manuscript (e.g., funding, provision of study materials, medical writing, article processing charges, etc.) **No time limit for this item.**	X___None	
		**Time frame: past 36 months**	
2	Grants or contracts from any entity (if not indicated in item #1 above).	X___None	
3	Royalties or licenses	X___None	
4	Consulting fees	X___None	
5		X___None	

	Payment or honoraria for lectures, presentations, speakers bureaus, manuscript writing or educational events		
6	Payment for expert testimony	X___None	
7	Support for attending meetings and/or travel	X___None	
8	Patents planned, issued or pending	X___None	
9	Participation on a Data Safety Monitoring Board or Advisory Board	X___None	
10	Leadership or fiduciary role in other board, society, committee or advocacy group, paid or unpaid	X___None	
11	Stock or stock options	X___None	
12	Receipt of equipment, materials, drugs, medical writing, gifts or other services	X___None	
13	Other financial or non-financial interests	X___None	

Please place an "X" next to the following statement to indicate your agreement:

X_ I certify that I have answered every question and have not altered the wording of any of the questions on this form.

ICMJE DISCLOSURE FORM

Date:31/03/2025
Your Name Peter Loughenbery
Manuscript Title **Paradoxical Activation of Entheseal Myeloid Cells by JAK1 and TYK2 Inhibitors via IL10 Antagonism**
Manuscript number (if known):____

In the interest of transparency, we ask you to disclose all relationships/activities/interests listed below that are related to the content of your manuscript. "Related" means any relation with for-profit or not-for-profit third parties whose interests may be affected by the content of the manuscript. Disclosure represents a commitment to transparency and does not necessarily indicate a bias. If you are in doubt about whether to list a relationship/activity/interest, it is preferable that you do so.

The following questions apply to the author's relationships/activities/interests as they relate to the current manuscript only.

The author's relationships/activities/interests should be defined broadly. For example, if your manuscript pertains to the epidemiology of hypertension, you should declare all relationships with manufacturers of antihypertensive medication, even if that medication is not mentioned in the manuscript.

In item #1 below, report all support for the work reported in this manuscript without time limit. For all other items, the time frame for disclosure is the past 36 months.

		Name all entities with whom you have this relationship or indicate none (add rows as needed)	Specifications/Comments (e.g., if payments were made to you or to your institution)
		Time frame: Since the initial planning of the work	
1	All support for the present manuscript (e.g., funding, provision of study materials, medical writing, article processing charges, etc.) **No time limit for this item.**	X____None	
		Time frame: past 36 months	
2	Grants or contracts from any entity (if not indicated in item #1 above).	X____None	
3	Royalties or licenses	X____None	
4	Consulting fees	X____None	
5		X____None	

	Payment or honoraria for lectures, presentations, speakers bureaus, manuscript writing or educational events		
6	Payment for expert testimony	__X__None	
7	Support for attending meetings and/or travel	___X_None	
8	Patents planned, issued or pending	_X___None	
9	Participation on a Data Safety Monitoring Board or Advisory Board	__X__None	
10	Leadership or fiduciary role in other board, society, committee or advocacy group, paid or unpaid	__X__None	
11	Stock or stock options	__X__None	
12	Receipt of equipment, materials, drugs, medical writing, gifts or other services	_X___None	
13	Other financial or non-financial interests	__x__None	

Please place an "X" next to the following statement to indicate your agreement:

X_ I certify that I have answered every question and have not altered the wording of any of the questions on this form.

ICMJE DISCLOSURE FORM

Date:31/03/2025
Your Name:__ Robert Dunsmuir
Manuscript Title **Paradoxical Activation of Entheseal Myeloid Cells by JAK1 and TYK2 Inhibitors via IL10 Antagonism**
Manuscript number (if known):____

In the interest of transparency, we ask you to disclose all relationships/activities/interests listed below that are related to the content of your manuscript. "Related" means any relation with for-profit or not-for-profit third parties whose interests may be affected by the content of the manuscript. Disclosure represents a commitment to transparency and does not necessarily indicate a bias. If you are in doubt about whether to list a relationship/activity/interest, it is preferable that you do so.

The following questions apply to the author's relationships/activities/interests as they relate to the current manuscript only.

The author's relationships/activities/interests should be defined broadly. For example, if your manuscript pertains to the epidemiology of hypertension, you should declare all relationships with manufacturers of antihypertensive medication, even if that medication is not mentioned in the manuscript.

In item #1 below, report all support for the work reported in this manuscript without time limit. For all other items, the time frame for disclosure is the past 36 months.

		Name all entities with whom you have this relationship or indicate none (add rows as needed)	Specifications/Comments (e.g., if payments were made to you or to your institution)
		**Time frame: Since the initial planning of the work**	
1	All support for the present manuscript (e.g., funding, provision of study materials, medical writing, article processing charges, etc.) **No time limit for this item.**	_x__None	
		**Time frame: past 36 months**	
2	Grants or contracts from any entity (if not indicated in item #1 above).	__x__None	
3	Royalties or licenses	x___None	
4	Consulting fees	_x__None	
5		_x__None	

	Payment or honoraria for lectures, presentations, speakers bureaus, manuscript writing or educational events		
6	Payment for expert testimony	__X__None	
7	Support for attending meetings and/or travel	___X_None	
8	Patents planned, issued or pending	X____None	
9	Participation on a Data Safety Monitoring Board or Advisory Board	__X__None	
10	Leadership or fiduciary role in other board, society, committee or advocacy group, paid or unpaid	__X__None	
11	Stock or stock options	__X__None	
12	Receipt of equipment, materials, drugs, medical writing, gifts or other services	X____None	
13	Other financial or non-financial interests	__x__None	

Please place an "X" next to the following statement to indicate your agreement:

X_ I certify that I have answered every question and have not altered the wording of any of the questions on this form.

ICMJE DISCLOSURE FORM

Date:31/03/2025
Your Name: Vishal Borse
Manuscript Title **Paradoxical Activation of Entheseal Myeloid Cells by JAK1 and TYK2 Inhibitors via IL10 Antagonism**
(if known):____

In the interest of transparency, we ask you to disclose all relationships/activities/interests listed below that are related to the content of your manuscript. "Related" means any relation with for-profit or not-for-profit third parties whose interests may be affected by the content of the manuscript. Disclosure represents a commitment to transparency and does not necessarily indicate a bias. If you are in doubt about whether to list a relationship/activity/interest, it is preferable that you do so.

The following questions apply to the author's relationships/activities/interests as they relate to the current manuscript only.

The author's relationships/activities/interests should be defined broadly. For example, if your manuscript pertains to the epidemiology of hypertension, you should declare all relationships with manufacturers of antihypertensive medication, even if that medication is not mentioned in the manuscript.

In item #1 below, report all support for the work reported in this manuscript without time limit. For all other items, the time frame for disclosure is the past 36 months.

		Name all entities with whom you have this relationship or indicate none (add rows as needed)	Specifications/Comments (e.g., if payments were made to you or to your institution)
		Time frame: Since the initial planning of the work	
1	All support for the present manuscript (e.g., funding, provision of study materials, medical writing, article processing charges, etc.) **No time limit for this item.**	_x___None	
		Time frame: past 36 months	
2	Grants or contracts from any entity (if not indicated in item #1 above).	_x___None	
3	Royalties or licenses	_x___None	
4	Consulting fees	_x___None	
5		_x___None	

	Payment or honoraria for lectures, presentations, speakers bureaus, manuscript writing or educational events		
6	Payment for expert testimony	__X__None	
7	Support for attending meetings and/or travel	X____None	
8	Patents planned, issued or pending	X____None	
9	Participation on a Data Safety Monitoring Board or Advisory Board	__X__None	
10	Leadership or fiduciary role in other board, society, committee or advocacy group, paid or unpaid	__X__None	
11	Stock or stock options	__X__None	
12	Receipt of equipment, materials, drugs, medical writing, gifts or other services	__x__None	
13	Other financial or non-financial interests	__x__None	

Please place an "X" next to the following statement to indicate your agreement:

X_ I certify that I have answered every question and have not altered the wording of any of the questions on this form.
