# Supplementary material for: Paradoxical Activation of Entheseal Myeloid Cells by JAK1 and Tyk2 Inhibitors via Interleukin‐10 Antagonism
Source: Arthritis Rheumatol. 2025 Jul 4;77(10):1373–86. doi: 10.1002/art.43210 (PMC12479184; doi:10.1002/art.43210)
Supplement: Supplementary file 2 — Appendix S1: Supplementary Information [file ART-77-1373-s001.docx]

**Methods- supplementary data**

**Luciferase assay**

THP1 cells stably transduced with a NF-κB-driven luciferase reporter were pretreated with 1 µM upadacitinib 1 hour prior to stimulation with 10 ng/ml LPS. Luciferase activity was then measured each hour after LPS stimulation (0-5 hr). Luciferase activity was measured by addition of Dual-Glo substrate and buffer solution (Promega) according to the manufacturer’s instructions, allowing 20 minutes of incubation time (RT), followed by measurement of luminosity by a Cytation 5 multimode plate reader (Agilent BioTek).

**Flow cytometry**

For intracellular analysis of cytokines, cells were treated with GolgiPlug (BD Bioscience; 1 µl per 10^6 cells) 8 hours prior to analysis. Cells were stained for cell viability (Zombie Fixable Dyes, BioLegend, 1:1000, 20-minute incubation) prior to blocking with 10% mouse serum and 1% human IgG for 10 minutes. Cells were then stained with surface marker antibodies (Table S1) at concentrations indicated by the manufacturers for 30 minutes in staining buffer (PBS, 1 mM EDTA, 2% BSA with BD brilliant stain buffer). For analysis of intracellular cytokines, after surface staining cells were fixed and permeabilized using BD Cytofix/Cytoperm fixation/permeabilization kit according to the manufacturer’s instructions and then stained with intracellular cytokine antibodies for 30 minutes (Table S1). Stained cells were analyzed on a Beckman-Coulter Cytoflex LX cytometer.

For analysis of phosphorylated STAT1, cells were fixed in 4% paraformaldehyde for 10 minutes at 37°C immediately after stimulation. Cells were then permeabilized using BD Phosflow Perm Buffer III for 30 minutes on ice. After fixation, cells were resuspended in staining buffer and anti-pSTAT1 (Table S1) for 1 hour. Cells were analyzed on a Beckman-Coulter Cytoflex LX cytometer.

**Bulk RNA sequencing and analysis**

RNA was extracted from cell pellets (~5x10^5^ cells) using the Total RNA Purification Kit (Norgen Biotek, Canada). Sequencing libraries were prepared, and sequenced (PE150, 9 Gb per sample) on the Illumina NovaSeq X plus at Novogene (Novogene, UK). Following quality control (removal of low-quality reads and reads containing adapters and poly-N), reads were aligned to the reference genome using Hisat 2 v2.0.5. featureCounts v1.5.0-p3 was used to count reads and calculate Fragments Per Kilobase of transcript per Million base pairs sequenced (FPKM). Differential expression analysis was performed with DESeq2 (DESeq2RPackage 1.20.0), and adjusted p-values (pAdj) calculated using the Benjamin and Hochberg’s approach. Genes with a pAdj ≤ 0.05 were assigned as differentially expressed. The clusterProfiler R package was used to test the statistical enrichment of differentially expressed genes in KEGG pathways, with pathways with corrected p values < 0.05 considered significantly enriched. Gene set enrichment analysis (GSEA) for KEGG pathways was performed using the GSEA analysis tool <http://broadinstitute.org/gsea/index.jsp>.

**Cytokine measurement**.

Cytokines were measured either by ELISA (IL-23, TNFα; Thermofisher) or by bead-based multiplexed immunoassay (LEGENDplex^TM^, BioLegend) according to the manufacturer’s instructions.

**Statistical analysis.**

GraphPad Prism software (La Jolla, CA, USA) was employed with ANOVA employment to calculate significance and Dunnet’s test used for multiple comparisons. Specific statistical tests are described in the corresponding figure legends. Significant differences between control and test with p values less than 0.05 were denoted with * as indicated in the figure legends or labelled with the exact p value where greater than 0.05 yet trends are evident. Any specific statistical tests are outlined in the figure legends.

**Table S1**

| Target | Fluorochrome | Clone | Catalogue ID |
| --- | --- | --- | --- |
| pSTAT1 (pY701) | Alexa-Fluor 647 | 4a | BD biosciences 612597 |
| CD3 | BUV395 | SK7 | BD biosciences 564001 |
| CD45 | APC / PE-Cy7 | HI30 | BD biosciences 560973 / 557748 |
| IL-17A | BV605 | BL168 | BioLegend 512326 |
| TNFα | PE-CF594 | MAb11 | BD biosciences 562784 |
| CD19 | BUV395 | SJ25C1 | BD biosciences 563549 |
| HLA-DR | BV421 | G46-6 | BD biosciences 562804 |
| CD14 | BV605 | M5E2 | BioLegend 301834 |
| CD80 | APC | 2D10.4 | eBiosciences 17-0809-42 |
| CD163 | PE | GHI/61 | BD biosciences 556018 |
| CD209 | FITC | 9E9A8 | BioLegend 330104 |
| IL-10Rα | NA | 37607 | R&D systems MAB274 |
| IL-19 | NA | Poly-clonal | R&D systems AF1035 |
| IFNAR1 | NA | MA5-42010 | Thermofisher MA5-42010 |

**Table S1:** **Antibodies utilized in flow cytometry panels and inhibition assays**

**Supplementary Figure Legends**


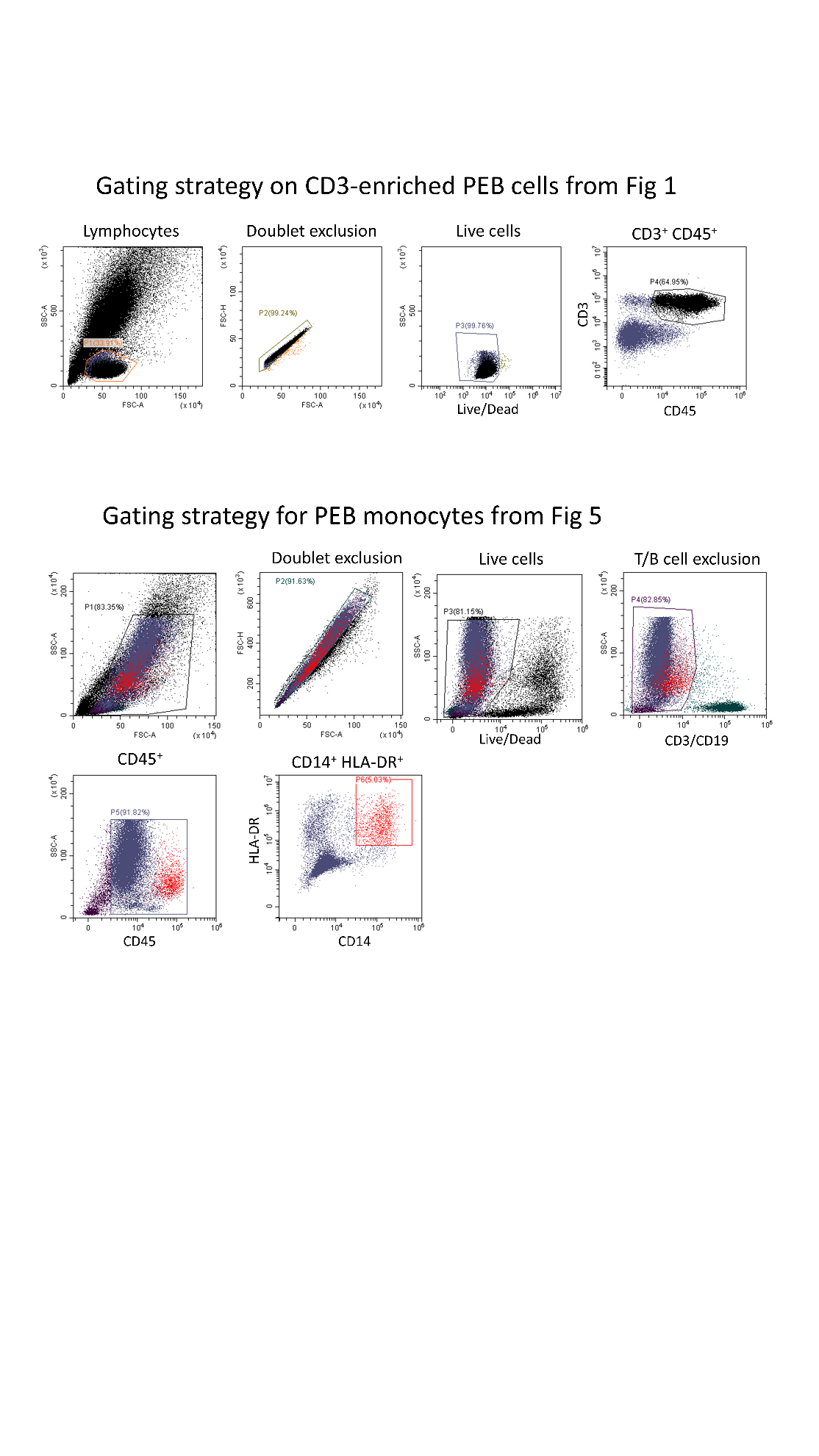


**Figure S3: Gating strategy;** Flow cytometry gating strategy used in figures 1 and 5

**
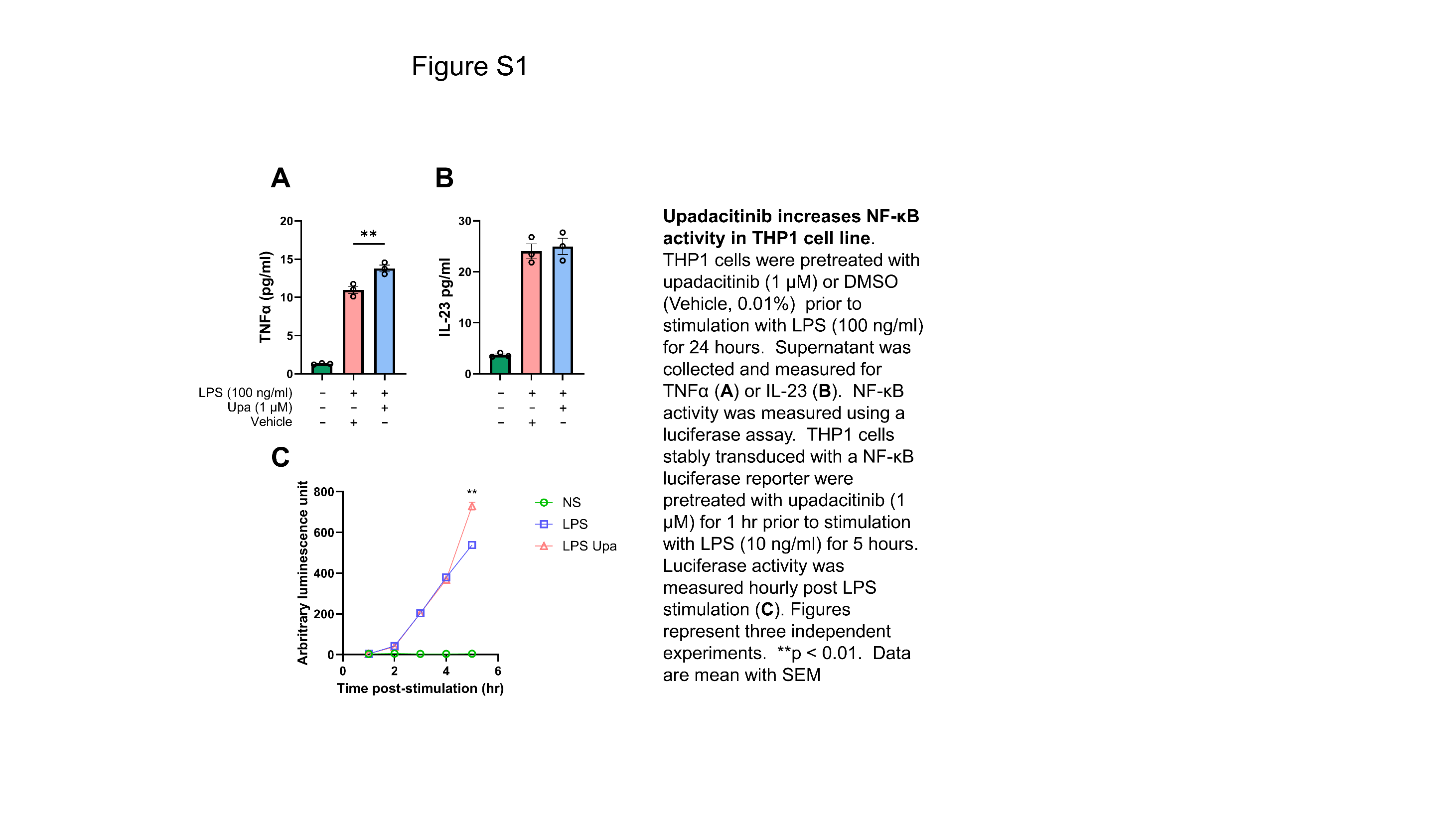
**

**Figure S2: Upadacitinib increases NF-κB activity in THP1 cell line.** THP1 cells were pretreated with upadacitinib (1 μM) or DMSO (Vehicle, 0.01%) prior to stimulation with LPS (100 ng/ml) for 24 hours. Supernatant was collected and measured for TNFα (A) or IL-23 (B). NF-κB activity was measured using a luciferase assay. THP1 cells stably transduced with a NF-κB luciferase reporter were pretreated with upadacitinib (1 μM) for 1 hr prior to stimulation with LPS (10 ng/ml) for 5 hours. Luciferase activity was measured hourly post LPS stimulation (C). Figures represent three independent experiments. **p < 0.01. Data are mean with SEM

**
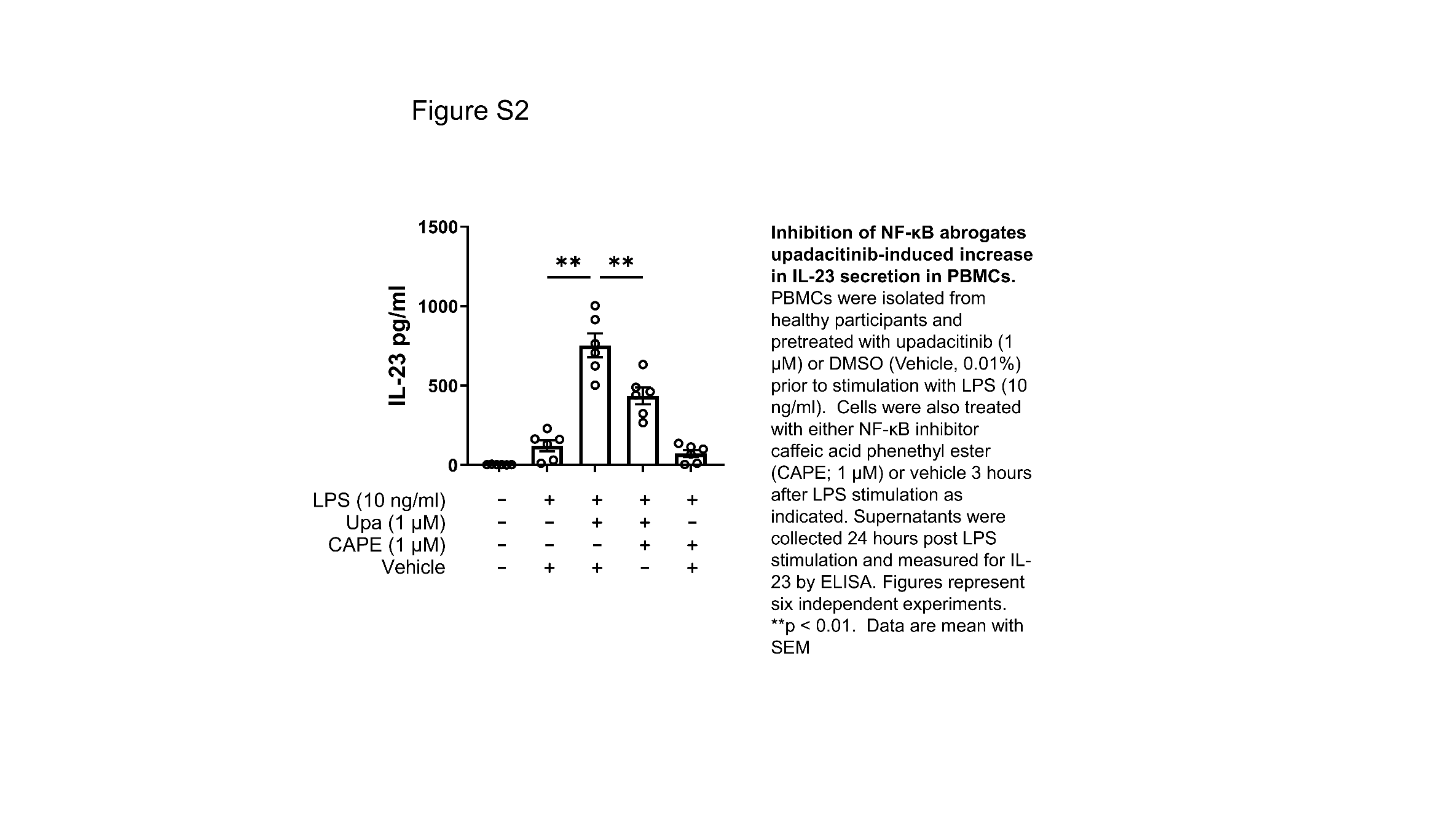
**

**Figure S3: Inhibition of NF-κB abrogates upadacitinib-induced increase in IL-23 secretion in peripheral blood leukocytes.** Peripheral blood leukocytes were isolated from healthy participants and pretreated with upadacitinib (1 μM) or DMSO (Vehicle, 0.01%) prior to stimulation with LPS (10 ng/ml). Cells were also treated with either NF-κB inhibitor caffeic acid phenethyl ester (CAPE; 1 μM) or vehicle 3 hours after LPS stimulation as indicated. Supernatants were collected 24 hours post LPS stimulation and measured for IL-23 by ELISA. Figures represent six independent experiments. **p < 0.01. Data are mean with SEM


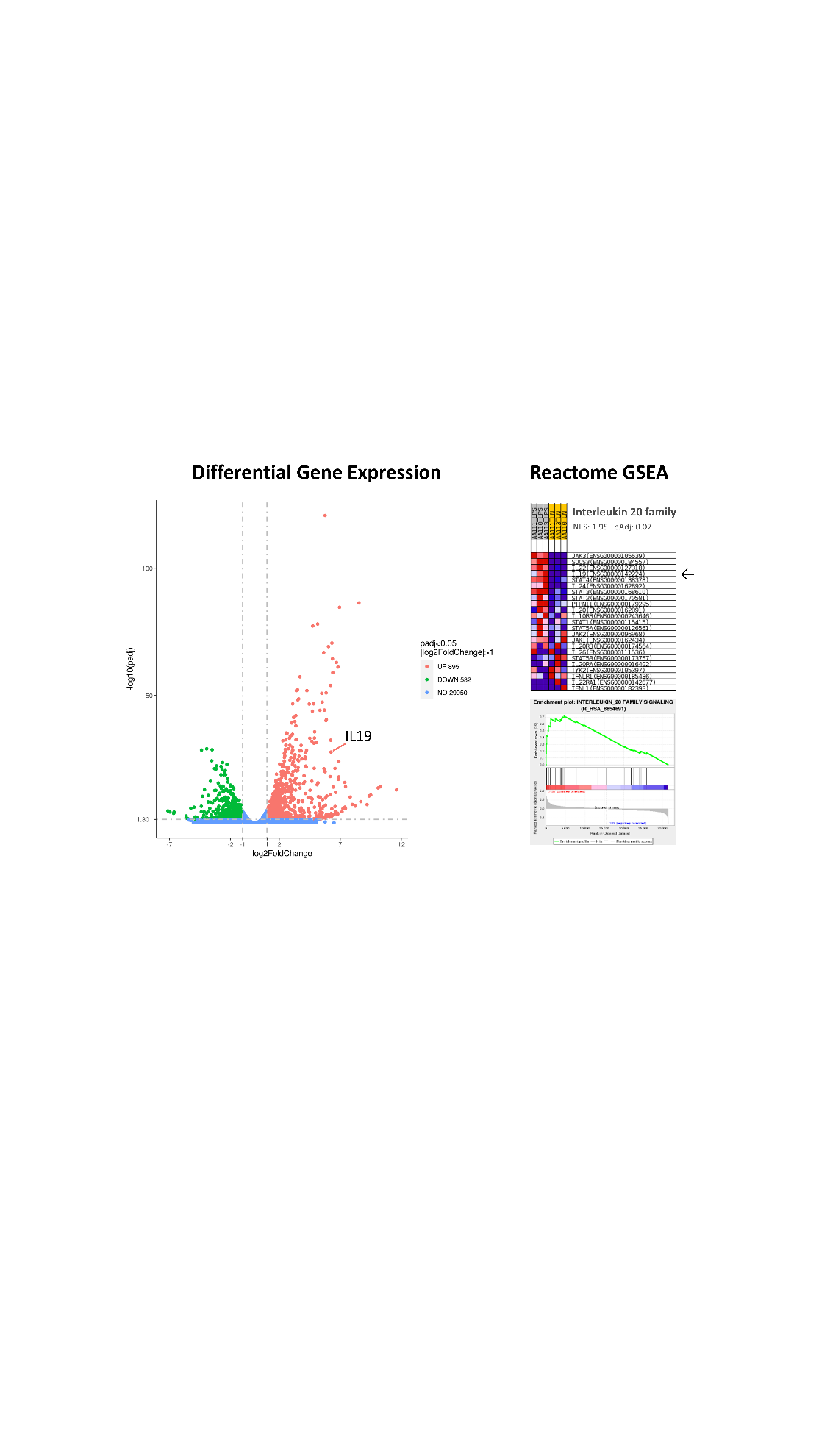


**Figure S4: RNA sequencing data indicates IL-19 signaling active following LPS stimulation;** A) Volcano plot displaying differentially expressed genes from RNASeq data between entheseal cells treated with LPS (10 ng/ml) for 12 hours and entheseal cells left untreated.  IL19 is annotated.  Green indicates downregulated in LPS vs untreated.  Red indicates upregulated in LPS vs untreated. B) Gene set enrichment analysis (GSEA) of the interleukin-20 reactome pathway showing normalized enrichment score (NES), expression heatmap for leading edge genes in the gene set, and GSEA plot. Arrow indicates IL19. Data are representative of three independent experiments. pAdj calculated using Benjamin and Hochberg’s approach.
